# Minimally Invasive Injectable Gel for Local Immunotherapy of Liver and Gastric Cancer

**DOI:** 10.1002/advs.202405935

**Published:** 2024-08-08

**Authors:** Xinghui Si, Guofeng Ji, Sheng Ma, Zichao Huang, Taiyuan Liu, Zhiyuan Shi, Yu Zhang, Jia Li, Wantong Song, Xuesi Chen

**Affiliations:** ^1^ Key Laboratory of Polymer Ecomaterials Changchun Institute of Applied Chemistry Chinese Academy of Sciences Changchun 130022 China; ^2^ Jilin Biomedical Polymers Engineering Laboratory Changchun 130022 China; ^3^ Department of General Surgery, Xuanwu Hospital Capital Medical University Beijing 100053 China; ^4^ School of Applied Chemistry and Engineering University of Science and Technology of China Hefei 230026 China; ^5^ The Second Hospital of Jilin University Changchun 130000 China

**Keywords:** cancer immunotherapy, gastric cancer, hydrogels, liver cancer, minimally invasive injection

## Abstract

Local immunotherapy represents a promising solution for preventing tumor recurrence and metastasis post tumor surgical resection by eliminating residue tumor cells as well as eliciting tumor‐specific immune responses. Minimally invasive surgery has become a mainstream surgical method worldwide due to its advantages of aesthetics and rapid postoperative recovery. Unfortunately, the currently reported local immunotherapy strategies are mostly designed to be used after open laparotomy, which go against the current surgical philosophy of minimally invasive therapy and is not suitable for clinical translation. Aiming at this problem, a minimally invasive injectable gel (MIGel) is herein reported loaded with immunotherapeutic agents for gastric and liver cancer postoperative treatment. The MIGel is formed by crosslinking between oxidized dextran (ODEX) and 4‐arm polyethylene glycol hydroxylamine (4‐arm PEG‐ONH_2_) through oxime bonds, which can be injected through a clinic‐used minimally invasive drainage tube and adhered tightly to the tissue. The loaded oxaliplatin (OxP) and resiquimod (R848) can be released constantly over two weeks and resulted in over 75% cure rate in orthotopic mouse gastric and liver cancer model. Collectively, a concept of minimally invasive local immunotherapy is proposed and MIGel is designed for local intraperitoneal cancer immunotherapy through minimally invasive surgery, with good clinical translation potential.

## Graphical abstract

A minimally invasive injectable gel formed by crosslinking between oxidized dextran and 4‐arm PEH‐ONH_2_ is designed for cancer postoperative treatment, which can be injected through minimally invasive drainage tubes and adhered tightly to the tissue. The loaded oxaliplatin and resiquimod can be released constantly and resulted in over 75% cure rate in orthotopic mouse gastric and liver cancer model.

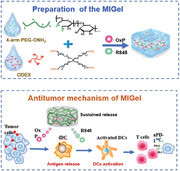

## 1 Introduction

Surgery is still the first choice for current cancer therapy, especially for solid tumors.^[^ ^1^ ^]^ However, the curative effects of surgical resections are limited due to the subsequent relapse from residual tumor cells as well as metastatic tumor niches. Postoperative local immunotherapy is quickly emerging as a consensus for preventing tumor postoperative recurrence.^[^ ^2^ ^]^ By placing biodegradable implants, films, patches, or in situ spray hydrogels at the resection site along with surgery, these means could eliminate residue tumor cells as well as elicit tumor‐specific immune responses, thus significantly prolong the survival time of patients post‐surgery.^[^ ^3^ ^]^

With the continuous development of minimally invasive surgical techniques, minimally invasive surgery represented by laparoscopic and robotic surgery, has become a mainstream surgical method worldwide due to its advantages of aesthetics, less bleeding, and rapid postoperative recovery. Unfortunately, the currently reported biodegradable materials‐based local immunotherapy strategies are mostly designed to be used after open laparotomy, which go against the current surgical philosophy of minimally invasive therapy and is not suitable for clinical translation. Furthermore, the above‐mentioned large‐scale implants or hydrogels are insufficient to overcome gastrointestinal peristalsis in dynamic and wet physical environments, which may shed from tissues and cause severe complications after surgery. Ideal materials meeting the requirements of minimally invasive operation should keep in a liquid form in the drainage tube while quickly gelation after contact with the tissue, tightly adhere with the tissue to overcome gastrointestinal peristalsis, and release the loaded cargos constantly to reduce side effects and establish immune memory effects.^[^ ^4^ ^]^ However, therapeutic materials meeting these requirements are still lacking.

**Scheme 1:**
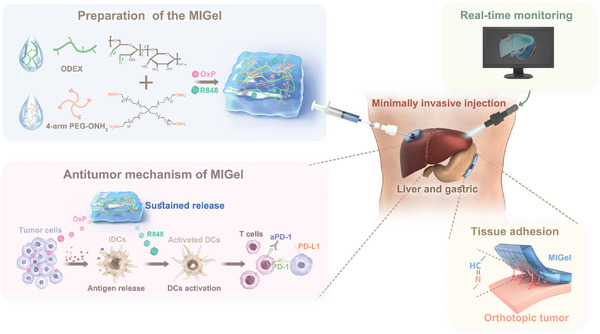
Schematic illustration of the preparation and mechanism of MIGel for orthotopic liver and gastric tumor therapy. The MIGel is cross‐linked by 4‐arm PEG‐ONH_2_ and ODEX. With the assistance of laparoscopy, the MIGel was minimally invasive injected through microcatheter. After the injection of MIGel, instantaneous adhesion to the surface of tissues was occurred due to the interaction between the aldehyde group of the material and the amino group on the surface of the tissue. The entire surgical process is being monitored in real‐time. The released OxP could induce tumor cell death thus promoting the release of antigens. Subsequently, the antigens were captured by immature DCs (iDCs) and presented to naïve T cells. Meanwhile, effector T cells mobilize to the tumor bed and kill tumor cells with the help of the separately injected aPD‐1 antibody.

Herein, we report a minimally invasive injectable gel (MIGel) with in situ gelation and tissue adhesion properties for post operation immunotherapy through minimally invasive surgery. The MIGel was fabricated by crosslinking between oxidized dextran (ODEX) and 4‐arm polyethylene glycol hydroxylamine (4‐arm PEG‐ONH_2_) through oxmide bonds (**Scheme** **1**). The formed gels could be injected through a clinic‐used minimally invasive catheters, while quickly stabilize when meeting the amines on the tissue and adhere to the tissue regardless of gastrointestinal peristalsis. We further loaded a drug combination of oxaliplatin (OxP) and resiquimod (R848) in the gel and proved that this strategy was much effective in preventing tumor postoperative relapse in mouse orthotopic gastric and liver cancer model. Our designed MIGel provides a valuable and clinically‐relevant option for local intraperitoneal cancer immunotherapy through minimally invasive surgery and possesses good clinical translation potential.

## 2 Results and Discussion

### 2.1 The Construction and Characterization of MIGel

**Figure 1:**
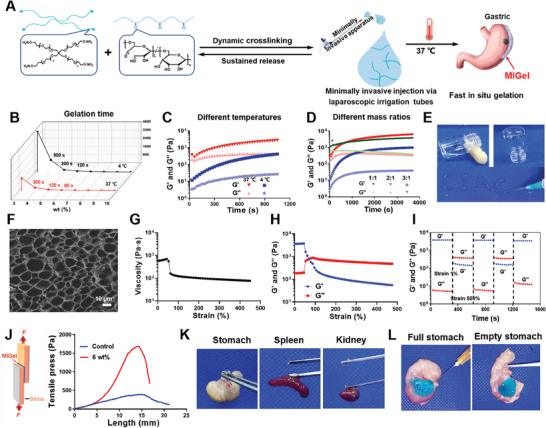
Construction and characterization of MIGel. A) Schematic illustration of the MIGel construction process. B) The gelation time of MIGels at different concentrations at 4 °C and 37 °C. C,D) Rheology properties of gels formed at different temperatures, and mass ratios. (E) The optical images of gels via a tube inverted test at 37 °C; The MIGel can pass through the needle (29 G), and then CIAC was drawn. F) SEM images of lyophilized MIGel. Scale bar: 10 µm. G) Viscosity of the MIGel at the strain of 1% to 500%. H) Shear stress sweep tests of the 6% MIGel at 37 °C. I) Self‐healing ability of the 6% MIGel evaluated with an alternating strain of 1% and 500% at room temperature. J) Tensile strength of MIGel between mouse skins and MIGel. K) Adhesion to various tissues including stomach, spleen, and kidney. L) The images of MIGel adhered to full and empty stomach.

The condensation reactions between aldehyde groups and amines including primary amine, hydroxylamine, and hydrazide are widely used cross‐linking approaches for hydrogel formation.^[^ ^5^ ^]^ In our previous reports, we designed implants or gels cross‐linked with imine bonds for local delivery of immune drugs.^[^ ^6^ ^]^ However, the implants or gels with poor shear thinning ability were not suitable for minimally invasive injection. In addition, the imine linkages are hydrolyzed much faster in aqueous solutions than oximes and hydrazones.^[^ ^7^ ^]^ Therefore, we used the oximes as the cross‐linking bonds in this study for MIGel construction (**Figure** **1A**). The purity of the 4‐arm PEG‐ONH_2_ modification was first verified by ^13^C Nuclear Magnetic Resonance (NMR) spectra analysis, the emergence of a peak at 74.8 ppm and the disappearance of a peak at 61.8 ppm highlight the completely modified of hydroxyl to hydroxylamine (Figure S1, Supporting Information).

For achieving minimally invasive injection, the hydrogels should have good injectability and adhesion after arriving at the tissue surface. Therefore, the gelation time is a key factor in designing the hydrogel, as too fast gelation is not beneficial for the injection, while too slow gelation is not conducive to the residence of gels at the lesion site.^[^ ^8^ ^]^ We first explored the influence of total concentrations of PEG‐ONH_2_ and ODEX on the gelation time. It can be observed that the gelation time decreased from 1200 s to 10 s as the total mass fraction (w/v) increased from 3% to 10% at 37 °C (Figure 1B). Considering that the temperature may affect the speed of gelation, the MIGel gelation time was further explored at 4 °C. The results showed that the gelation speed at 4 °C was much slower than that at 37 °C, suggesting that MIGel could be easily minimally invasive injected at low temperature while quickly gel once contacting with the tissue in vivo. We finally chose the 6% (w/v) mass fraction with a gelation time of 120 s at 37 °C and 300 s at 4 °C for the following study. This difference in gelation time could be explained by the mechanical strength at different temperatures, as the storage modulus (G′) and loss modulus (G’″) were both much higher at 37 °C than that at 4 °C in 18 mins (Figure 1C). The mechanical strength of the hydrogel is a key factor in determining the degradation time.^[^ ^9^ ^]^ Hence, we further explored the influence of mass ratios of PEG‐ONH_2_ and ODEX on the mechanical strength (G′, G″) of the formed MIGel. As shown in Figure 1D, when the mass ratios of 4‐arm PEG‐ONH_2_ to ODEX increased from 1/1 to 3/1, the mechanical strength first increased and then decreased. At the mass ratio of 2/1, the MIGel exhibited the highest G′ of over 6000 pa. Mass fraction of 6% and mass ratio of 2/1 were finally selected as the formulation for the preparation of MIGel. Scanning electron microscopy (SEM) images showed the porous and interconnected structure of the lyophilized gel, with pore sizes ranging from 10 to 30 µm (Figure 1F).

The good shear‐thinning and self‐healing ability is a guarantee for realizing minimally invasive injection. Hence, we performed these tests in the following study. Stress sweep assay was performed to determine the linear viscoelastic region of the MIGel (Figure 1G). As expected, the viscosity of the MIGel dramatically decreased when the strain increased from 1% to 100%, suggesting that the hydrogel crosslinked with oxime dynamic covalent bonds exhibited good shear‐thinning properties. Moreover, the MIGel could be continuously injected to draw the letter “CIAC” without clogging, further highlighting their shear thinning properties (Figure 1E). The rapid recovery of gel strength following injection is important for in vivo applications of hydrogels. The strain sweep test and step‐strain measurement were performed on the MIGel to examine its self‐healing properties. As shown in Figure 1H,G′ of the MIGel decreased to lower than G′′ at a strain of 70%, and G′ immediately dropped to less than 100 Pa at a strain of 200%, indicating the disruption of the hydrogel network at high strain. When the strain was switched to a low value (1%), the MIGel showed complete recovery of G′ and G′′ within seconds (Figure 1I). In addition, the disruption‐recovery process was reproducible and could be repeated without deterioration of the mechanical properties of the MIGel, suggesting the good self‐healing ability.

For minimally invasive surgery, good adhesion is important to prevent the MIGel from tearing off the fixed site. Hence, we performed an adhesion test for the formed hydrogels. As shown in Figure 1J, the 6% MIGel has 1500 Pa adhesion strength between the skins and the MIGel. We also tested the adhesion of MIGel on various organs. The results showed that the MIGel could be adhering to tissue organs of mice including stomach, spleen, and kidney (Figure 1K). Excitingly, the MIGel can overcome gastric peristalsis. No matter whether it is gastric filling or gastric emptying after feeding, the MIGel can still be firmly adhered to the stomach in 1 day (Figure 1L). All the results demonstrated the minimally invasive feasibility of MIGel and good adhesion to the tissue.

### 2.2 The Degradation, Minimally Invasive Injectability, Drug Release, and Safety Evaluation of MIGel

**Figure 2:**
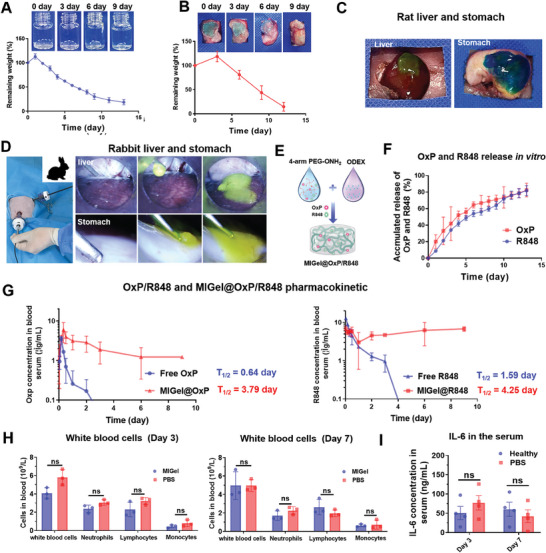
The degradation, injectability and drug release, and safety evaluation of MIGel. A) In vitro degadation of MIGel under physiological conditions (PBS, pH 7.4). The white circles represent the remaining hydrogels during degradation period. B) In vivo degradation of MIGel after injecting on the surface of gastric. C) The images of MIGel injected on the liver and stomach of rats though the syringe with 29 G needle. D) The images of MIGel injected on the liver and stomach of rabbits though laparoscopic procedure. E) The schematic for the preparation of MIGel@OxP/R848. F) The accumulated release profiles of OxP and R848 in vitro. G) In vivo pharmacokinetic profiles following the intraperitoneal injection of free OxP/R848 or MIGel@OxP/R848 injected on the gastric of rats. H) The complete blood count after injection of MIGel on the stomach at day 3 and day 7. I) The IL‐6 concentration in the serum after injection of MIGel on the stomach at day 3 and day 7. Data are the mean ± standard deviation (*n = 3*). *ns*, not significant.

Next, we assessed the degradation of the MIGel in vitro and in vivo. The MIGel were placed in phosphate buffered saline (PBS, pH 7.4) and incubated at 37 °C with constant shaking. The weights of the MIGel were measured at a predetermined time points. It can be observed that the MIGel first experience a swelling and then gradually degraded, and the weight percent decreased to 18.5% in 11 days (**Figure** **2A**). For in vivo degradation evaluation, 300 µL gels were injected onto the stomach of BALB/c mice, and residual gels were collected and photographed at defined time points. As shown in Figure 2B, in vivo degradation of the MIGel lasted for over 12 days. More importantly, the MIGel was observed continuously adhered on the surface of stomach, further suggested the persistent adhesion to the tissue surface. Next, we attempted minimally invasive injection in rats, and the MIGel was injected onto the liver and stomach through the syringe. It can be observed that the MIGel could form a layer of gel close and adhere to these organs (Figure 2C). To further explore the clinical translational potential of MIGel, the feasibility of minimally‐invasive intraperitoneal injection was performed in the larger animal rabbit model. Anesthesia was maintained with an inhalational anesthetic. After a pneumoperitoneum was established, the MIGel was injected topically to the stomach or liver with a long needle through a laparoscopic trocar. Interestingly, ultrafast gelation in seconds with superior tissue adhesion was observed after injection (Figure 2D; Movie S1, Supporting Information). This accelerated gelation property after contacting with the tissue would be much helpful for the administration through a minimally invasive injection way, and the reason for this performance may be due to the cross‐linking of aldehyde groups in the MIGel with the amines on the tissue surface proteins.^[^ ^10^ ^]^ More excitingly, after over 4 h observation, the MIGel could still adhere to the organs without any deformation, fully proved that the MIGel can ignore gastrointestinal peristalsis in practical applications.

OxP, as the first‐line chemotherapeutic drug for the treatment of gastric cancer, is reported to induce immunogenic cell death (ICD) in many cancer cell types, and further boost the tumor‐associated antigen release.^[^ ^11^ ^]^ R848, a small molecular Toll‐like receptor 7/8 (TLR7/8) agonist stimulating the secretion of type I interferons (IFNs) from the innate immune cells, could stimulate the activation of myeloid DCs and plasmacytoid DCs.^[^ ^12^ ^]^ The combination of OxP and R848 could play as an in situ vaccine for activating anti‐tumor immunity. Therefore, we chose the combination of OxP and R848 to be loaded inside the MIGel for following gastric cancer therapy. OxP (300 µg) and R848 (150 µg) were easily loaded into the MIGel by simple mix during the preparation process of MIGel (Figure 2E). The release of loaded therapeutic agents from the MIGel was investigated then. As shown in Figure 2F, a gradual and stable release of OxP and R848 from the MIGel was observed at similar rate, with 82% of OxP and 80% of R848 released in 13 days, respectively. The sustained release of OxP and R848 is mainly speculated as the following two points: (1) during the gel formation process, the aldehyde groups of ODEX could interact with the amino groups of R848, thereby attaching the drug to the polymer material and enabling sustained drug release along with the degradation. (2) the sustained release of OxP could be attributed to the coordination interaction between the carboxyl groups produced by the excessive oxidation of dextran and the platinum. The gradual release of the therapeutic agents from the gels ensured sustained stimulation of the immune cells at around the injection site, which would benefit the establishment of effective immune responses.^[^ ^13^ ^]^

Furthermore, the pharmacokinetics of free OxP/R848 and MIGel@OxP/R848 (OxP: 35 mg kg^−1^, R848 17.5 mg kg^−1^) were assessed in rats. At pre‐determined time points (0, 1 h, 2 h, 4 h, 8 h, 12 h, day 1, day 2, day 3, day 6, day 9, day 12, day 15), blood samples were collected for determination the concentrations of OxP and R848. As shown in Figure 2G, OxP and R848 in MIGel showed significantly prolonged blood circulation half‐life (t_1/2_ = 3.79 day, 4.25 day) compared to free OxP and R848 (t_1/2_ = 0.64 day, 1.59 day). The area under the curve (AUC_0‐T_) of the MIGel@OxP/R848 group was 10 times and 4 times higher than that of free OxP and R848 groups, respectively (Table S1, Supporting Information). This steady drug concentration could avoid too high initial drug concentration in causing toxic and side effects, as well as too low end‐stage drug concentrations hardly exerting the therapeutic effects.

The safety of the MIGel was assessed by MTT assay. Murine 3T3 cells and gastric cancer MFC cells were incubated with free OxP and MIGel, and the cell viability was measured after 48 h. 3T3 and MFC cell viability was over 80% after 48 h incubation with the MIGel even at a concentration of 30 mg mL^−1^, suggesting good biocompatibility of the materials (Figure S2, Supporting Information). The cytotoxic of OxP and R848 loaded MIGel was further assessed. As shown in Figure S3 (Supporting Information), MIGel@OxP/R848 exhibited weaker tumor cell inhibiting ability compared to free OxP/R848 due to the gradual release. We further used BALB/c mice to assess the systemic inflammatory response. After being treated with PBS or MIGel, the blood cells and concentrations of inflammatory cytokine IL‐6 were monitored. Results showed that there were no remarkable changes of white blood cells including neutrophils, lymphocytes, and monocytes at day 3 and day 7 (Figure 2H), and no obvious changes in the IL‐6 levels (Figure 2I). In addition, the mice treated with MIGel did not show any sign of weight loss (Figure S4, Supporting Information), and the livers after contact with the MIGel were observed at different time points. As shown in Figure S5 (Supporting Information), the MIGel did not cause significant changes in the liver tissue or notable inflammation throughout the observation period. All these investigations evidenced the good safety of the MIGel for in vivo application.

### 2.3 MIGel for Subcutaneous Gastric Tumor Therapy

**Figure 3:**
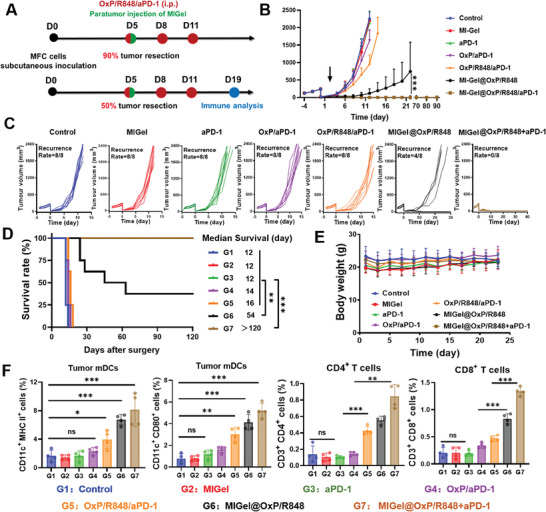
In vivo therapeutic efficacy on subcutaneous gastric tumor resection model. A) Treatment scheme for subcutaneous gastric tumor model (90% tumor resection) and immune analysis (50% tumor resection). B) Average tumor growth curves after operation. *n = 8*. C) Individual gastric tumor growth curves after surgical operation. D) Survival curves of mice in various treatment groups. *n = 8*. (E) The body weight changes of the mice during the treatment period. *n = 8*. (F) The analysis of activated DCs (CD11c^+^MHCII^+^ and CD11c^+^CD80^+^), CD4^+^ T cells, and CD8^+^ T cells in the tumor at day 19. *n = 4*. Data are presented as mean ± standard error on the mean (S.D.) *p < 0.05, **p < 0.01, and ***p < 0.001*. ns*, not significant.

To test the therapeutic effects of MIGel on post‐surgery therapy, we first established an subcutaneous incomplete tumor resection model by excising ≈90% of the tumor when MFC tumors on the C57BL/6 mice reached a volume of 200 – 300 mm^3^. The mice were randomly divided into 7 groups: untreated (Control, G1), invasive injectable gels without drug (MIGel, G2), aPD‐1 (G3), free OxP/aPD‐1 (G4), free OxP/R848/aPD‐1 (G5), MIGel@OxP+aPD‐1 (G6), MIGel@OxP/R848+aPD‐1 (G7), and residual tumor growth was constantly monitored (**Figure** **3A**). As shown in Figure 3B–D, tumor relapses appeared rapidly in untreated mice, and the median post‐surgery survival time was only 12 days. No therapeutic effects were observed in the MIGel‐treated and aPD‐1 treated mice, with a median post‐surgery survival time of similar 12 days. The widely used clinical therapeutic schedule OxP/aPD‐1 and free drug combination OxP/R848/aPD‐1 showed moderate therapeutic effects and almost no benefit in prolonging the median survival time. In contrast, MIGel@OxP/R848 treatment could obviously inhibit the tumor relapse, and the median survival time was prolonged to 54 days with nearly half of the mice cured. Notably, the combination of MIGel@OxP/R848 and aPD‐1 could further enhance the therapeutic efficacy and resulted in complete tumor eradication. All the mice treated with MIGel@OxP/R848**+**aPD‐1 were cured and no tumor relapse appeared in the 120 days observation period. In addition, there was no significant body weight loss of the mice in all groups during the treatment period, indicating the safety of these treatments (Figure 3E). These data validated that the proper drug combination as well as the long‐term and sustained release of the drug is necessary for activating systemic antitumor immunity.

To further analyze the potential reasons for the therapeutic effects in various groups, we established another subcutaneous model to evaluate immune cells infiltration within the tumor after treatment. In this model, only 50% of the tumor was removed to ensure that a sufficiently large tumor was obtained for immune analysis at the end of the 14‐day treatment period. As shown in Figures 3F and S6–S10 (Supporting Information), the treatment of MIGel@OxP/R848 and MIGel@OxP/R848+aPD‐1 elicited significantly stronger DCs activation than other groups, suggesting the full activation of antitumor immunity induced by local and sustained release of drug combinations. Furthermore, increased numbers of CD4^+^ and CD8^+^ T cells were detected inside the tumors in the MIGel@OxP/R848+aPD‐1 treatment group, further suggesting the activation of local antitumor immunity.

### 2.4 MIGel for Treating Orthotopic Gastric Cancer

**Figure 4:**
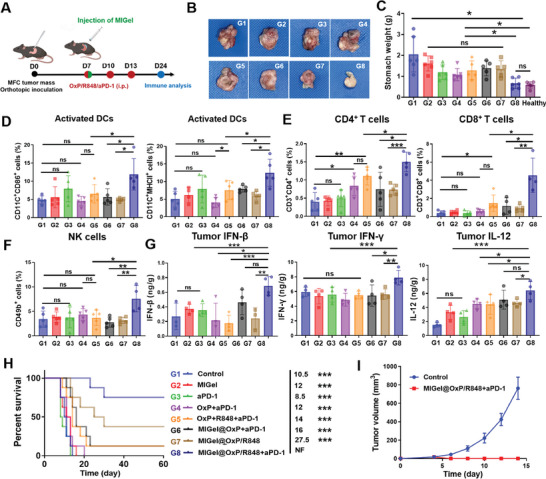
In vivo therapeutic efficacy on orthotopic gastric cancer model and immune analysis after various treatments. A) Treatment scheme. B) The representative images of the excised stomachs after various treatments (day 24). C) The stomach weights after various treatments. *n = 6*. D) The proportions of activated DCs in tumors after various treatments at day 24. *n = 5*. E) Tumoral CD4^+^ and CD8^+^ T cells after various treatments at day 24. *n* = 5. F) The proportions of NK (CD49b^+^) cells in tumors after various treatments at day 24. *n* = *5*. G) The level of IFN‐β, IFN‐γ, and IL‐12 cytokines in tumors after various treatments. *n* = *5*. H) Survival curves of mice after various treatments. *n = 8*. I) Rechallenge tumor growth curves of naïve mice and the survival mice after 60 days of MIGel@OxP/R848+aPD‐1 treatment. *n = 6*. *p < 0.05, **p < 0.01, and ***p < 0.001. *ns*, not significant.

To further investigate the tumor therapy potency of the MIGel, another round of therapy was assessed on the orthotopic MFC gastric cancer model, which is closer to the clinical gastric cancer (**Figure** **4A**). One week after tumor mass inoculation, mice were randomly divided into eight groups and treated with Control (G1), MIGel (G2), aPD‐1 (G3), free OxP/aPD‐1 (G4), free OxP/R848/aPD‐1 (G5), MIGel@OxP/R848 (G6), MIGel@OxP+aPD‐1 (G7), MIGel@OxP/R848+aPD‐1 (G8). Mice were sacrificed on day 24, and the stomach was excised for tumor evaluation. Similar to the therapeutic effect observed in subcutaneous model, MIGel@OxP/R848+aPD‐1 treatment exhibited the best therapeutic efficacy. No obvious tumor niches were observed on the excised stomach, and the stomach weight was almost the same to the healthy stomach (Figure 4B,C). In addition, the body weights of the mice were not significantly impacted by the treatments (Figure S11, Supporting Information).

To reveal the mechanism of antitumor effects induced by MIGel@OxP/R848, we measured the infiltrated immune cells and inflammatory cytokines in the tumors after various treatments. As shown in Figures 4D–G and S12–S19 (Supporting Information), much increased activated DCs, CD4^+^ T cells, CD8^+^ T cells, and NK cells with correspondingly elevated levels of interferon‐β (IFN‐β), IFN‐γ and IL‐12 were observed in the tumor tissues in the MIGel@OxP/R848+aPD‐1 group. This result suggested the necessity of localized ICD‐inducing chemotherapy, immune adjuvant R848 in combination with immune check point inhibitor aPD‐1 antibody. The plasmacytoid DCs (pDCs) are activated and secrete type I interferon (IFN‐I) for priming and activating natural killer (NK) cells to kill tumor cells,^[^ ^14^ ^]^ myeloid DCs (mDCs) are activated and secrete interleukin 12 (IL‐12) for further priming and activating T cells.^[^ ^15^ ^]^ All the results confirmed that robust innate and acquired antitumor immune responses were triggered by local chemoimmunotherapy with the help of the MIGel.

The therapeutic effect of the drug combination was further validated by the survival experiment. 75% of the mice in the MIGel@OxP/R848+aPD‐1 treatment group were survivable during the 60 day observation period (Figure 4D). To test the immune memory effect, a tumor rechallenge experiment was performed on the surviving mice after 60 days in the MIGel@OxP/R848+aPD‐1 treatment group with the same MFC tumor cells re‐inoculated (1 × 10^6^ cells per mouse) to the left flank. Notably, in contrast to quick tumor growth on healthy mice, the cured mice in the MIGel@OxP/R848+aPD‐1 group could prevent tumor recurrence‐(Figure 4E). This result clearly proved that the MIGel@OxP/R848+aPD‐1 treatment could elicit tumor‐specific memory effects and prevent tumor relapse.

### 2.5 RNA‐Seq Analysis for Treatment Mechanism of Drug Combinations

**Figure 5:**
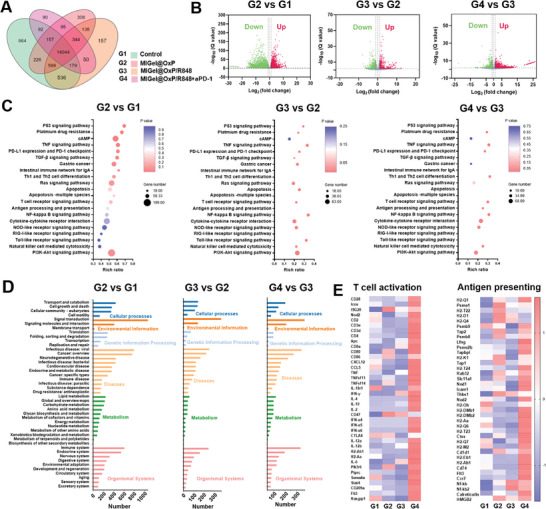
The whole genome RNA‐seq for gastric tumors after various treatments. A) Venn interaction of control (G1), MIGel@OxP (G2), MIGel@OxP/R848 (G3) and MIGel@OxP/R848+aPD‐1 (G4). *n = 3*. B) The volcano plots illustrating differentially regulated gene expression from RNA‐seq analysis between the control and MIGel@OxP, MIGel@OxP and MIGel@OxP/R848, MIGel@OxP/R848 and MIGel@OxP/R848+aPD‐1. Upregulated and downregulated genes are shown in red and green, respectively. *n = 3*. C) KEGG pathway analysis of changed genes between the control and MIGel@OxP, MIGel@OxP and MIGel@OxP/R848, MIGel@OxP/R848 and MIGel@OxP/R848+aPD‐1. *n = 3*. D) KEGG functional clustering of genes that were changed for biological processes. E) Heatmaps of differentially expressed genes related to T cells activation, antigen processing, and presentation. *n = 3*. (*p < 0.05, **p < 0.01, and ***p < 0.001).

Inspired by the exciting therapeutic effect on orthotopic gastric cancer model, we further explored the action mechanism of the drug combination by whole genome RNA‐seq. The venn relationships among the Control, MIGel@OxP, MIGel@OxP/R848, and MIGel@OxP/R848+aPD‐1 groups were shown in **Figure** **5A**. After MIGel@OxP treatment, 9862 genes exhibited differential expression (*p*‐value < 0.05 and fold change ≥1) compared to the control group, including 4190 upregulated genes (red dots) and 5672 downregulated genes (green dots). Furthermore, the application of R848 caused 2640 gene differential expressions with 1452 upregulated and 1188 downregulated (MIGel@OxP/R848 versus MIGel@OxP). When combined with aPD‐1 antibody, 2700 genes exhibited differential expression with 1769 upregulated and 940 downregulated (MIGel@OxP/R848+aPD‐1 versus MIGel@OxP/R848) (Figure 5B). Subsequently, the main pathways of the regulated genes were assessed by Kyoto Encylopaedia of Genes and Genomes (KEGG) pathway enrichment. As shown in Figure 5C, the changed genes in the MIGel@OxP group compared to control group were mainly involved in p53 signaling pathway, platinum drug resistance, cAMP signaling pathway, and TNF signaling pathway. However, with the addition of R848, the changed genes were mainly involved in the NF‐κB, TNF signaling pathway, Toll‐like receptor signaling pathway, and so on, suggesting the immune adjuvant role as the R848 as a TLR7/8 agonist.^[^ ^16^ ^]^ Furthermore, after the combination with aPD‐1, significantly different genes were observed in the pathway of TNF, NF‐κB, Th1, and Th2 cell differentiation, T cell receptor, Toll‐like receptor, PD‐L1 and PD‐1 checkpoint pathway, antigen processing, and presentation, fully proving the immune activation induced by the drug combinations. In addition, the role of changed genes was mainly associated with cellular processing, environmental information pathway, genetic information processing, diseases, metabolism, and organismal systems (Figure 5D). The genes associated with T cell activation, antigen processing, and presentation were further assessed. Notably, the gene heatmaps showed that obviously upregulated genes associated with T cell activation, antigen processing, and presentation in the MIGel@OxP/R848+aPD‐1 group were observed compared to that in other groups (Figure 5E), which was consistent with the flow cytometry analysis of immune cells infiltration in the tumor. Taken together, these data demonstrated that this drug combination is necessary for efficiently stimulating the immune responses to facilitate cancer immunotherapy.

### 2.6 MIGel for Treating Hepatic Metastases and Orthotopic Liver Cancer

**Figure 6:**
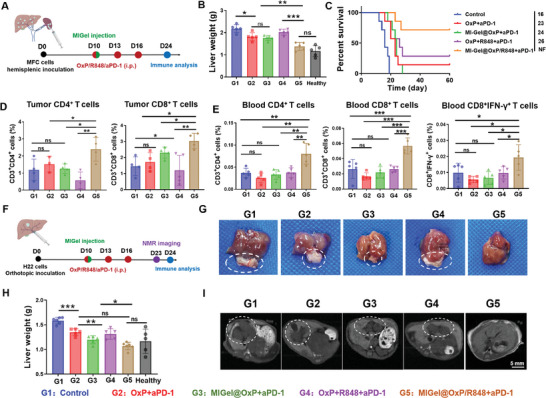
In vivo therapeutic efficacy on liver metastasis model and orthotopic liver cancer model. A) Treatment scheme for liver metastasis model. B) The liver weights after various treatments. *n = 5*. C) Survival curves of mice after various treatments. *n = 7*. D) Tumoral CD4^+^ and CD8^+^ T cells after various treatments at day 24. *n = 4‐5*. E) The proportions of CD4^+^, CD8^+^, and IFN‐γ^+^ T cells in blood after various treatments at day 24. *n* = *4*‐*5*. F) Treatment scheme for orthotopic liver model. G) The representative images of the excised livers after various treatments (day 24). The white circles represent the orthotopic liver tumors. H) The liver weights after various treatments. *n = 5*. I) The nuclear magnetic resonance images (T1) of the livers in various treatment groups. The white circles represent the orthotopic liver tumors. *p < 0.05, **p < 0.01, and ***p < 0.001. *ns*, not significant.

Among the common intra‐abdominal tumors, the liver cancer is also often resected and treated through laparoscopic minimally invasive surgery in clinic.^[^ ^17^ ^]^ Hence, the anti‐cancer effect of the MIGel was further assessed on the hepatic metastases model and orthotopic liver cancer model. For the establishment of hepatic metastases model from gastric tumor, 2 × 10^6^ MFC cells were inoculated by hemisplenic injection.^[^ ^18^ ^]^ 10 days after inoculation, mice were randomly divided into 5 groups: Control (G1), free OxP/aPD‐1 (G2), MIGel@OxP+aPD‐1 (G3), free OxP/R848/aPD‐1 (G4), MIGel@OxP/R848+aPD‐1 (G5) (**Figure** **6A**). While free OxP/aPD‐1, MIGel@OxP**+**aPD‐1, and free OxP/R848/aPD‐1 could only partially delay the tumor growth, the MIGel@OxP/R848+aPD‐1 treatment could markedly inhibit the metastatic liver tumor growth with similar liver weight to that of healthy mice (Figure 6B). As a result, 75% of mice in the MIGel@OxP/R848+aPD‐1 treatment group survived for over 60 days (Figure 6C). For exploring the therapeutic mechanism, the local and systemic immune responses were assessed by flow cytometry. As shown in Figures 6D and S20–S23 (Supporting Information), enhanced CD4^+^ and CD8^+^ T cells infiltration in the tumor was observed after MIGel@OxP/R848+aPD‐1 treatment. In addition, the highest proportions of CD4^+^, CD8^+^, and IFN‐γ^+^ T cells were detected in the blood of the MIGel@OxP/R848+aPD‐1 treatment group, suggesting the fully activated systemic antitumor immunity (Figure 6E).

For the establishment of orthotopic liver cancer model, 2 × 10^6^ H22 cells were injected directly on the liver of BABL/c mice.^[^ ^19^ ^]^ 10 days after inoculation, mice were randomly divided into 5 groups: Control (G1), free OxP/aPD‐1 (G2), MIGel@OxP+aPD‐1 (G3), free OxP/R848/aPD‐1 (G4), MIGel@OxP/R848+aPD‐1 (G5) (Figure 6F). On day 24, obvious liver tumor niches were observed in the control and free OxP/aPD‐1 treated group. The MIGel@OxP+aPD‐1 with no immune adjuvant R848 could achieve in moderately delaying the growth of liver tumors, but were far less efficient than that achieved in the MIGel@OxP/R848+aPD‐1 group (Figure 6G,H). The antitumor effect of MIGel was further demonstrated by nuclear magnetic resonance (NMR) imaging. As shown in Figure 6I, apparent tumors were observed in the liver in the control, OxP+aPD‐1, MIGel@OxP+aPD‐1, and free OxP/R848/aPD‐1 groups, while no tumor niches were observed in the MIGel@OxP/R848+aPD‐1 treatment group, suggesting obviously better tumor therapeutic effect. In addition, HE staining of livers was analyzed after various treatments. As shown in Figure S24 (Supporting Information), numerous clear nuclei and regular shapes of liver tumor cells were observed in control, OxP/aPD‐1, MIGel@OxP+aPD‐1, and free OxP/R848/aPD‐1 group. In contrast, no liver tumor cells were observed in MIGel@OxP/R848+aPD‐1, fully demonstrating that the therapy effectively inhibited othotopic liver tumor growth.

To find out the mechanism of antitumor effects induced by localized chemoimmunotherapy, we examined the proportions of T cells in the blood after various treatments by flow cytometry (Figures S25–S27, Supporting Information). It was found that the localized ICD‐inducing chemotherapy in combination with immune adjuvant R848 and immune checkpoint blockade antibody anti‐PD‐1 could effectively increase the frequency of systemic CD4^+^ and CD8^+^ T cells, suggesting the strong antitumor immune responses elicited by local and sustained delivery of drug combinations. All these results demonstrated that our proposed localized chemoimmunotherapy can be used to treat liver tumors and could also offer a strong immune memory effect, similar to that observed with other types of tumor models.

## 3 Conclusion

In this study, we report a MIGel loaded with OxP/R848 administered by laparoscopy and microcatheters for orthotopic gastric cancer, orthotopic and metastatic liver cancer therapy. The MIGel could be quickly transformed to the gel with strong adhesion after contact with the tissue and maintained in vivo for over 12 days, which is facile for clinical application during minimal operation. The loaded OxP and R848 could be gradually released from the MIGel, serving as in situ vaccines for stimulating antitumor immune responses. In vivo studies showed that this local therapy with the combination of aPD‐1 immune checkpoint inhibitor could completely inhibit post‐surgical tumor relapse in a subcutaneous gastric tumor model, significantly inhibit the growth of orthotopic gastric tumor with 75% tumor cure rate, and also inhibit tumor growth in orthotopic liver tumor and liver metastasis model. Based these results, we anticipate the application of this MIGel in future clinic tumor therapy.

## 4 Experimental Section

### The Characterization of the MIGel

The structure of the freeze‐dried MIGel was characterized by SEM (JSM‐7000F, JEOL Ltd, Tokyo, Japan). Rheological experiments were performed on a Physica MCR 301 Rheometer (Anton Par). The copolymer solution was placed between parallel plates of 25 mm diameter and with a fixed gap of 0.5 mm at 37 °C. The storage modulus (G′) and loss modulus (G″) were measured as functions of time. The outer edge of the sample was coated by a thin layer of silicon oil for preventing the evaporation of water during measurement. The tensile test was texted on a universal testing machine (AGS‐X 100N, SHIMADZU, Japan).

The MIGel was *in‐situ* formed between two mice skins (15 mm in length and 15 mm in width). Then MIGel or water was placed in between two mice skins, and the tensile stress was measured. The tensile rate was 1 mm min^−1^. All tests were repeated three times. For the adhesion of the MIGel to the tissue, 150 µL of the MIGel was injected onto the tissue and observed its adhesion by lifting the gel with tweezers.

### Safety Evaluation of the MIGel

For assessing the safety of the MIGel, Female C57BL/6 mice were randomly divided into 2 groups: only surgery (Sham) and blank gels (MIGel). At day 3 and 7 post injection and surgery, blood samples were collected for texting the number of blood cells by MEK‐6318 hematology analyzer and the level of IL‐6.

### In Vivo Anti‐Tumor Efficiency in MFC Subcutaneous Tumor Model

MFC cells (2 × 10^6^) cells were subcutaneously injected on the skin of the C57BL/6 mice to generate the MFC incomplete model. When the volume of subcutaneous tumors reached ≈200–300 mm^3^, the mice were anesthetized with isoflurane and fixed on an operating table, the abdominal skin was disinfected with alcohol. Then, a scalpel was used to remove ≈90% volume of the tumor, leaving ≈10% residual tumor tissue (≈20–30 mm^3^) behind in resection cavity to mimic the clinical condition of incomplete tumor resection. MIGel or solutions were injected on the bed of resectable cavity. Finally, the small incision was sewn together with 5‐0 absorbable suture and disinfected with 75% ethanol. The mice were kept warm and monitored carefully until conscious.

(1) $$Tumorvolumes\left(V_{t},mm^{3}\right)\;=a\times b^{2}/2$$

Body weights were measured every 2 days to evaluate systemic toxicity.

Mice with subcutaneous gastric tumors were randomly divided into 7 groups: Control, MIGel, OxP+aPD‐1, MIGel@R848+aPD‐1, free OxP+R848+aPD‐1, OxP+MIGel@R848+aPD‐1 and MIGel@OxP/R848+aPD‐1 (OxP, 22.5 mg kg^−1^; R848, 7.5 mg kg^−1^; aPD‐1, 15 mg kg^−1^). Free OxP, R848, or aPD‐1 was intraperitoneally (i.p.) injected every three days (in total 3 times). The MIGel was injected on the excised tumor by operation. The small incision was sewn together with 5‐0 silk stitching and disinfected with 75% ethanol. The mice were kept warm and monitored carefully until conscious. Tumor‐suppression‐rate (TSR%) was calculated using the following equations:

(2) $$TSR\%=\left[\left(T_{Wc}-T_{Wx}\right)/T_{Wc}\right]\times 100\%$$

T_Wc_ represents the average weight of tumors in the control group. T_Wx_ represents that in the treatment group.

For immune analysis infiltration within the tumors, another tumor suppression experiment was conducted. In this experiment, to ensure that a sufficiently large tumor sample was obtained for immune analysis at the end of the 14‐day treatment period, 50% of the tumor was only removed. After the treatment, tumors were harvested. The tumors were cut into small pieces and digested in tumor dissociation buffer. Then the supernatant from the digested tumor tissues was collected, filtered, centrifuged, and resuspended. Finally, the cell suspension is stained with fluorophore‐conjugated antibodies. The cells were then washed twice and analyzed using the BD FACS Celesta.

### In Vivo Anti‐Tumor Efficiency in MFC Orthotopic Tumor Model

The establishment of orthotopic gastric tumor was achieved by transplanting tumor masses from subcutaneous tumors. The subcutaneous gastric tumors (200 −300 mm^3^) were cut into many small pieces with about 10–20 mm^3^. The tumor pieces were washed with PBS and adhere to the surface of the stomach wall by medical glue. In the end, the small incision was sewn up with 5‐0 silk line and disinfected with 75% ethanol. The mice were kept warm and monitored carefully until conscious.

### For the Treatment of Orthotopic Gastric Tumors, the Mice were Randomly Divided into 8 Groups

Control, MIGel, aPD‐1, OxP+aPD‐1, MIGel@OxP+aPD‐1, free OxP+R848+aPD‐1, MIGel@OxP/R848 and MIGel@OxP/R848+aPD‐1 (OxP, 22.5 mg kg^−1^; R848, 7.5 mg kg^−1^; aPD‐1, 15 mg kg^−1^). Free OxP, R848, or aPD‐1 was intraperitoneally (i.p.) injected every three days (in total 3 times). The MIGel was injected on the gastric by operation.

At 17 days after the treatment, tumors and spleens were harvested. The tumors were cut into small pieces and digested in tumor dissociation buffer. Then the supernatant from the digested tumor tissues was collected, filtered, centrifuged, and resuspended. Spleen tissue was mechanically ground, filtered, and resuspended. Red blood cell (RBC) lysis buffer was used to lyse the erythrocytes. Finally, cell suspensions were stained with fluorophore‐conjugated antibodies. The cells were then washed twice and analyzed using BD FACS Celesta.

For the analysis of immune cells in the blood, the blood was collected, filtered, centrifuged, and resuspended at day 14. RBC lysis buffer was used to lyse the erythrocytes. Finally, the cell suspension is stained with fluorophore‐conjugated antibodies. The cells were then washed twice and analyzed using the BD FACS Celesta.

For the tumor rechallenge study, the mice in the MIGel@OxP/R848+aPD‐1 group survived for 60 days and untreated mice were subcutaneously injected 1 × 10^6^ MC38 cells at the left flank, and the tumor volume was recorded.

### In Vivo Anti‐Tumor Efficiency in Liver Metastasis Model and Orthotopic Liver Tumor Model

For the establishment of gastric tumor metastasis to liver model, healthy female C57BL/6 mice were narcotized routinely with isoflurane and fixed on an operation table. The abdominal skin was disinfected and a 1 cm incision was made in the middle of abdomen to expose spleen. After the spleen was cut to 2 segments by the electrotome, the 5 × 10^5^ MFC cells were administrated via portal vein of the spleen. The administrated half spleen was removed from the mice. In the end, the small incision was sewn up with 5‐0 silk line and disinfected with 75% ethanol. The mice were kept warm and monitored carefully until conscious.

For H22 orthotopic liver cancer model establishment, healthy female BALB/c mice were narcotized routinely with isoflurane and fixed on an operation table. The abdominal skin was disinfected and a 1 cm incision was made in the middle of abdomen to expose liver. H22 cells obtained from the ascites of KM mice were washed with PBS and diluted into a concentration of 2 × 10^7^ cells mL^−1^, subsequently 5 × 10^5^ cells (25 µL) were injected into the left lobe of the liver, and syringe pinhole was coagulated with electrotome immediately to prevent tumor cells leak into the abdominal cavity. In the end, the small incision was sewn up with 5‐0 silk line and disinfected with 75% ethanol. The mice were kept warm and monitored carefully until conscious.

After the establishment of orthotopic liver tumor model and the liver metastasis model, the mice were randomly divided into 5 groups, Control, free OxP/aPD‐1, MIGel@OxP+aPD‐1, free OxP/R848/aPD‐1, MIGel@OxP/R848+aPD‐1 (OxP, 22.5 mg kg^−1^; R848, 7.5 mg kg^−1^; aPD‐1, 15 mg kg^−1^). The MIGel was injected on the surface of liver by operation. The body weights were monitored during the treatment period. After the treatment, the tumor and the blood were collected for assessing the antitumor immunity mechanism.

At 13 days after the treatment, the orthotopic liver tumors of mice were analyzed by nuclear magnetic resonance imager (Israel, Aspect M7). At 14 days after the treatment, tumors were harvested. The analysis of immune cells in the tumor and the blood is consistent with the previous procedural steps.

### RNA Whole Sequencing Analysis

RNA‐seq sequencing analysis was performed on tumors from the orthotopic gastric cancer model after treatment (Control, BI@OxP, BI@OxP/R848, and BI@OxP /R848/aPD‐1). After 17 days of administering various treatments, tissue samples from orthotopic MFC tumor (100 mg) were sent to the Beijing Genomics Institute (BGI), China, for comprehensive whole‐genome RNA sequencing analysis. Initially, the raw sequencing data were subjected to quality control using trimmomatic software, which included the removal of low‐quality reads and adapter trimming. Subsequently, the cleaned reads were aligned to the reference genome using the Dr. TOM tool from BGI. For the alignment results, gene expression quantification was performed with feature Counts, utilizing transcripts per million (TPM) as the unit of expression. The identification of differentially expressed genes (DEGs) was carried out using DESeq2 software to select genes that exhibited significant differences in expression between the treatment groups and the control group.

### Statistical Analysis

The GraphPad Prism 8.0 Software was used to perform the statistical analysis. At least three times independent tests were performed on all experiments. The results were expressed as means ±standard deviation SD or mean ± standard error of the mean (SEM). Sample size (n) for each statistical analysis was added in each Figure legends. The Student's t‐test and one‐way analysis of variance (ANOVA) were used to analyze the statistical significances. A value of *p < 0.05, **p < 0.01, and ***p < 0.001 were judged to be statistically significant and very significant respectively. (*n.s*. = no significance)

### Ethical Statement

All animal studies were carried out according to the guidelines approved by the Animal Welfare and Ethics Committee of Changchun Institute of Applied Chemistry, Chinese Academy of Sciences (2022‐0026). Female C57BL/6 mice (6‐8 weeks), female BABL/c mice (6‐8 weeks), female SD rats (6‐8 weeks), and rabbits were purchased from Beijing Vital River Laboratory Animal Technology Co., Ltd. (Beijing, China). Mice were sacrificed and recorded as death when the tumor volume reached 2000 mm^3^.

## Conflict of Interest

The authors declare no conflict of interest.

## Supporting information

Supporting Information

Supplemental Movie 1

## Data Availability

The data that support the findings of this study are available from the corresponding author upon reasonable request.
