# Supplementary material for: Minimally Invasive Injectable Gel for Local Immunotherapy of Liver and Gastric Cancer
Source: Adv Sci (Weinh). 2024 Aug 8;11(38):2405935. doi: 10.1002/advs.202405935 (PMC11481255; doi:10.1002/advs.202405935)
Supplement: Supplementary file 1 — Supporting Information [file ADVS-11-2405935-s001.docx]

Supporting information for

**Minimally Invasive Injectable Gel for Local Immunotherapy of Liver and Gastric Cancer**

*Xinghui Si, Guofeng Ji, * Sheng Ma, Zichao Huang, Taiyuan Liu, Zhiyuan Shi, Yu Zhang, Jia Li, Wantong Song, * and Xuesi Chen*

**Materials and Methods**

**Materials**

Requimod (R848) and were purchased from Sigma-Aldrich (St. Louis, MO, USA). Anti-PD-1 antibody (aPD-1, Isotype: Rat IgG1, κ; Catalog: BE0031) was purchased from bioX Cell (NH, USA). Rat IgG (Catalog: SP032) was bought from Solarbio Science & Technology (Beijing, China). Oxaliplatin (OxP), sodium periodate and Dextran (Mw = 40 kDa) were purchased from Aladdin Chemical Reagent Company (Beijing, China). 4-arm PEG-ONH_2_ (Mw = 10 kDa) was purchased from JINGPI TECHNOLOGY Co. Ltd. (Beijing, China). All antibodies (**Table S2**) used for flow cytometry were purchased from BD biosciences (CA, USA) and bioLegend (CA, USA). IL-6, IL-12, TNF-α and IFN-β ELISA kits (**Table S3**) were purchased from Anoric biotechnology (Tianjin, China).

**Mice and cell lines**

Healthy BALB/c, C57BL/6 mice (female, 8 weeks old, 18-20 g), SD rats (female, 8 weeks old, 180-200 g) and New Zealand White rabbits (female, 20 weeks old, 4 kg) were purchased from Beijing Vital River Laboratory Animal Technology Co., Ltd. (Beijing, China). The animals were raised in a specific pathogen free (SPF) animal laboratory. MFC cells, 3T3 fibroblasts and H22 cells were purchased from Dalian meilunbio Technology Co., Ltd.

**ODEX synthesis and MIGel preparation:**

The ODEX was synthesized following the previously reported method.^[1]^ The amounts of sodium periodate were adjusted for obtaining 40% oxidation degree dextran. The oxidation degree means the percentage of oxidized glucose units in total units. To remove crude products, dialysis was conducted against deionized water for 3 days (molecular weight cut off = 3.5 kDa). The MIGel were prepared by cross-linking different ratios of PEG-ONH_2_ and ODEX by Schiff’s base reaction at the concentration of 6% (w/v). For example, 6 wt % 4-arm PEG-ONH_2_ and 6 wt % ODEX were dissolved in water, respectively. OxP was dissolved in the 4-arm PEG-ONH_2_ solution. R848 (150 μg) was firstly dissolved in DMSO (3 μL), and then added to 4-arm PEG-ONH_2_ solution. Then, 4-arm PEG-ONH_2_ and ODEX solution were mixed to obtain the MIGel.

**Cytotoxicity assay**

Murine gastric cancer MFC cells and 3T3 fibroblasts were cultured in Dulbecco’s modified Eagle’s medium (DMEM) with high glucose containing 10% fetal bovine serum (FBS), 1% penicillin and 1% streptomycin at 37 °C in a 5% CO_2_ atmosphere.

3T3 fibroblasts were used to test the biocompatibility of MIGel according to the following procedure. 3T3 cells were seeded into 96-well plates at a density of ~7000 cells per well, 3T3 cells were incubated overnight in 150 μL DMEM. After the fibroblasts cells had adhered to the culture dish, replace the medium with 200 μL fresh DMEM containing different concentrations of the MIGel. At predetermined time points, 20 μL of MTT was added to the 96-well plates for a further 3 h of incubation. Supernatants were then removed and 150 μL DMSO was added. After shaking for 5 min, absorbances were measured on a Bio-Rad 680 microplate reader at 490 nm. The relative cell viability (%) was calculated using following formula:

Cell viability (%) = (*A*_experimental_/*A*_control_) × 100

*A*_experimental_ and *A*_control_ represent the absorbance of the experimental and control wells, respectively. Data are the average ± SD (*n = 3*). The IC_50_ was defined as the concentration causing a 50% loss of cell proliferation.

***In vitro* and *in vivo* degradation analyses**

To investigate *in vitro* degradation, the MIGel was placed in phosphate buffered saline (PBS) buffer (pH = 7.4) and incubated at 37 ℃ with constant shaking at 90 rpm. Then, the residual weight of the MIGel was measured at the predetermined time points. For *in vivo* degradation evaluation, the MIGel was surgically injected on the gastric of BALB/c mice. At the defined points (1, 3, 5, 7, 9, 11, 13, 15 and 17 days), the mice were sacrificed and residual MIGel were collected and photographed.

***In vitro* release of OxP and R848 from the MIGel**

The release behavior of the MIGel was investigated *in vitro*. OxP or R848-loaded MIGel was placed in 2 mL pH 7.4 PBS in a shaking incubator (37°C, 90 rpm). At the defined time points, the release medium was removed and replaced with fresh medium. R848 concentrations were detected by UV-Vis spectrometry (λ = 300 nm, LAMBDA 365 UV/Vis Spectrophotometers). OxP concentrations were determined by Inductive Coupled Plasma Emission Spectrometer ([iCAP RQ ICP-MS](https://www.thermofisher.cn/cn/zh/home/industrial/mass-spectrometry/inductively-coupled-plasma-mass-spectrometry-icp-ms/single-quadrupole-inductively-coupled-plasma-mass-spectrometry-sq-icp-ms.html)).

**Pharmacokinetics**

SD rats (180-200 g) were randomly divided into 4 groups (free OxP/R848, MIGel@OxP/R848). Free OxP (35 mg kg^-1^) or R848 (17.5 mg kg^-1^) were intraperitoneally (i.p.) administered, respectively. MIGel@OxP/R848 at the same doses was injected on the gastric by surgery. At the defined time points (0, 1, 2, 4, 8, 12 h, 1, 2, 3, 6, 9, 12, 15, 18 day), blood samples were collected for determination the concentrations of OxP and R848. R848 concentrations were detected by high performance liquid chromatography (λ = 300 nm, the mobile phase system was Acetonitrile/water at the ratio of 2:1). OxP concentrations were determined by Inductive Coupled Plasma Emission Spectrometer. The half-life of the drug, area under the drug concentration–time curve from 0 to the corresponding time in plasma, total plasma clearance and apparent volume of distribution were calculated though the PKSolver.^[2]^

**Statistical analysis**

The GraphPad Prism 8.0 Software were used to perform the statistical analysis. At least three times independent tests were performed on all experiments. The results were expressed as means ±standard deviation SD or mean ± standard error of the mean (SEM). Sample size (n) for each statistical analysis was added in each Figure legends. The Student’s t-test and one-way analysis of variance (ANOVA) were used to analyze the statistical significances. A value of **p* < 0.05, ***p* < 0.01 and ****p* < 0.001 were judged to be statistically significant and very significant respectively. (n.s. = no significance)

**Supplemental Figures**


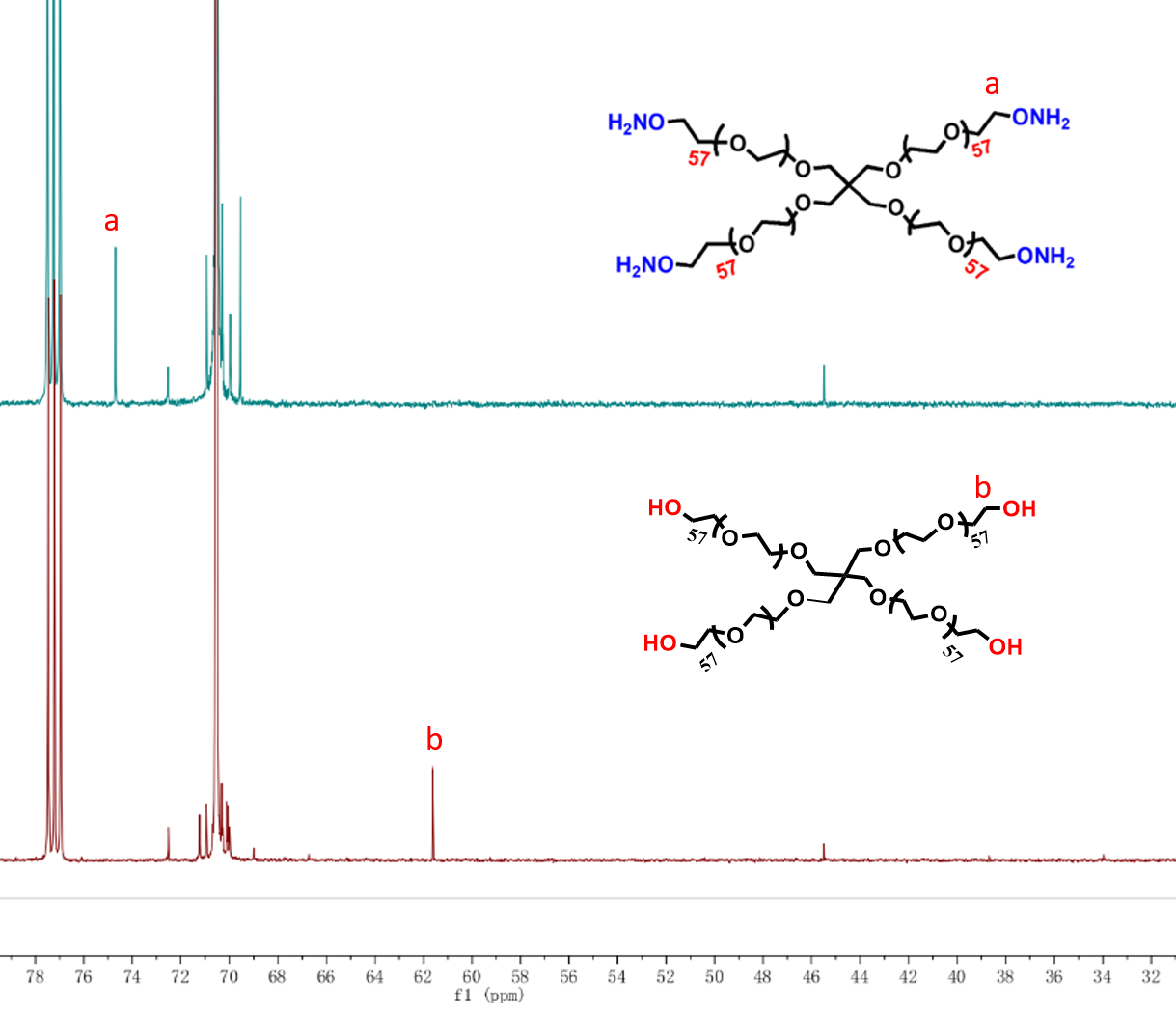


**Figure S1**. The ^13^C NMR spectra 4-arm PEG-OH and 4-arm PEG-ONH_2_.


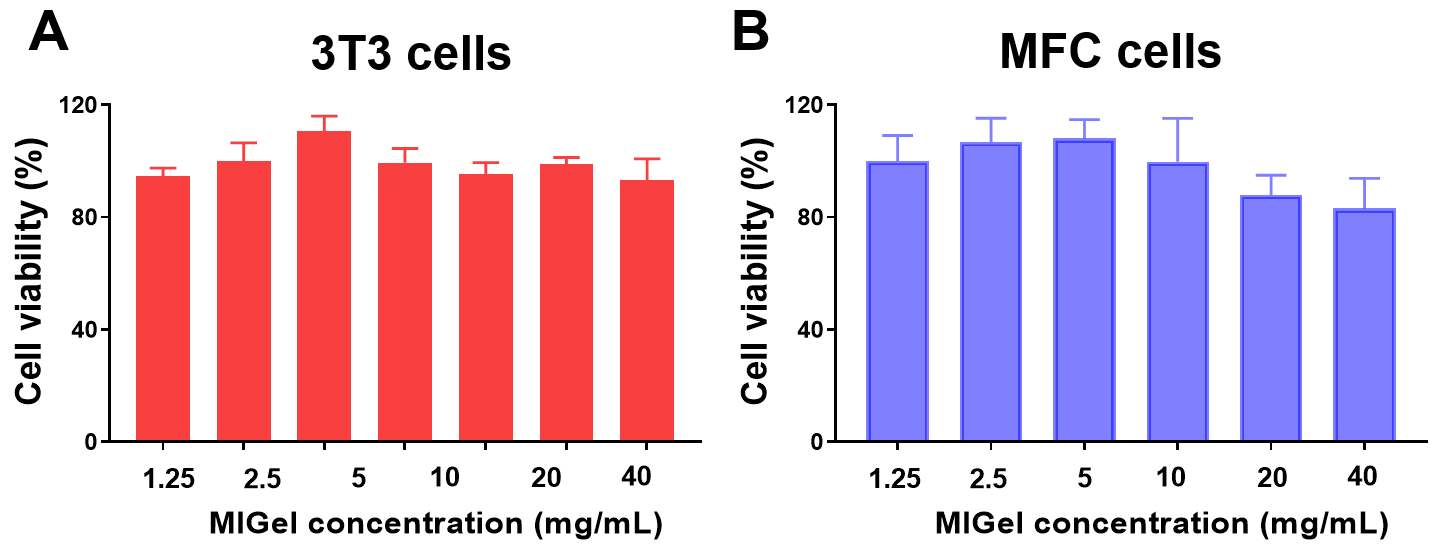


**Figure S2**. The cytotoxicity of MIGel on the 3T3 cells (A) and MFC cells (B) after incubation with various concentrations of MIGel for 72 h. *n=3.*


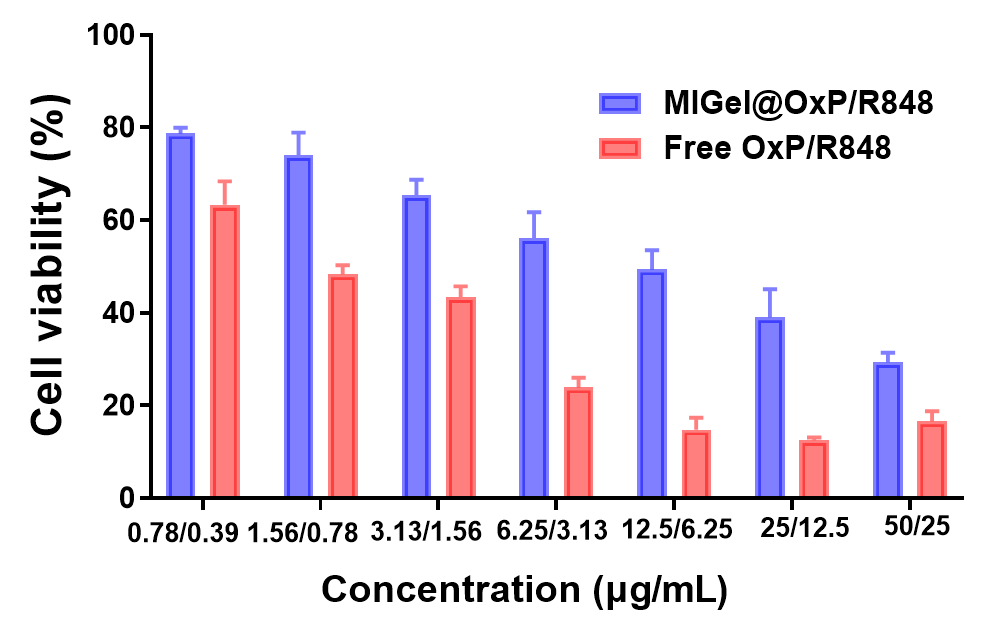


**Figure S3**. The cytotoxicity of MIGel@OxP/R848 and free OxP/R848 on the MFC cells at different concentrations after 72 h incubation. *n=3.*


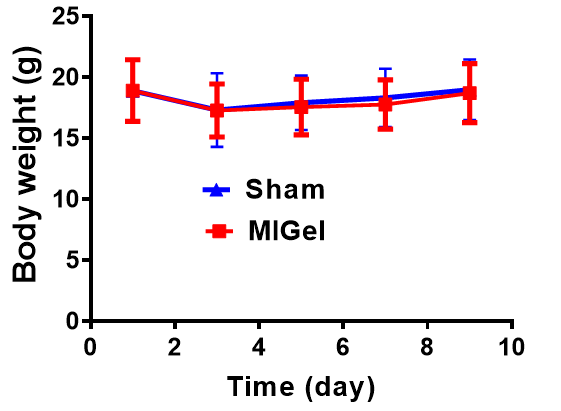


**Figure S4**. The changes of body weight after injection the MIGel at day 0 on the gastric of BABL/c mice or only surgery (Sham).


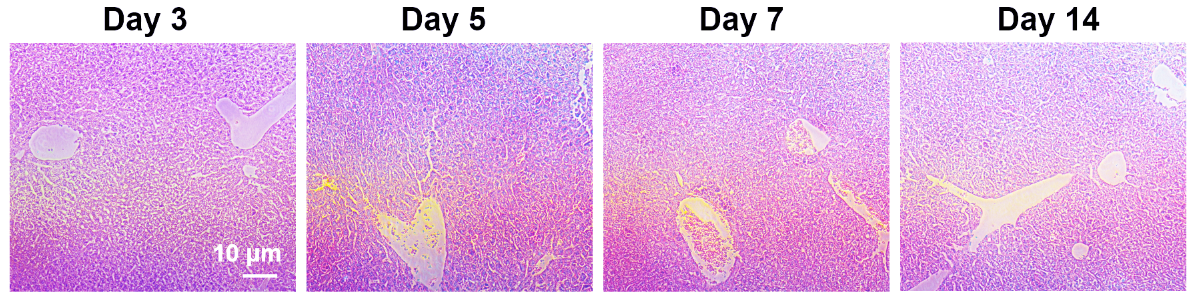


**Figure S5**. HE images of the livers after 300 μL MIGel injection on livers at different times (day 3, 5, 7,14).


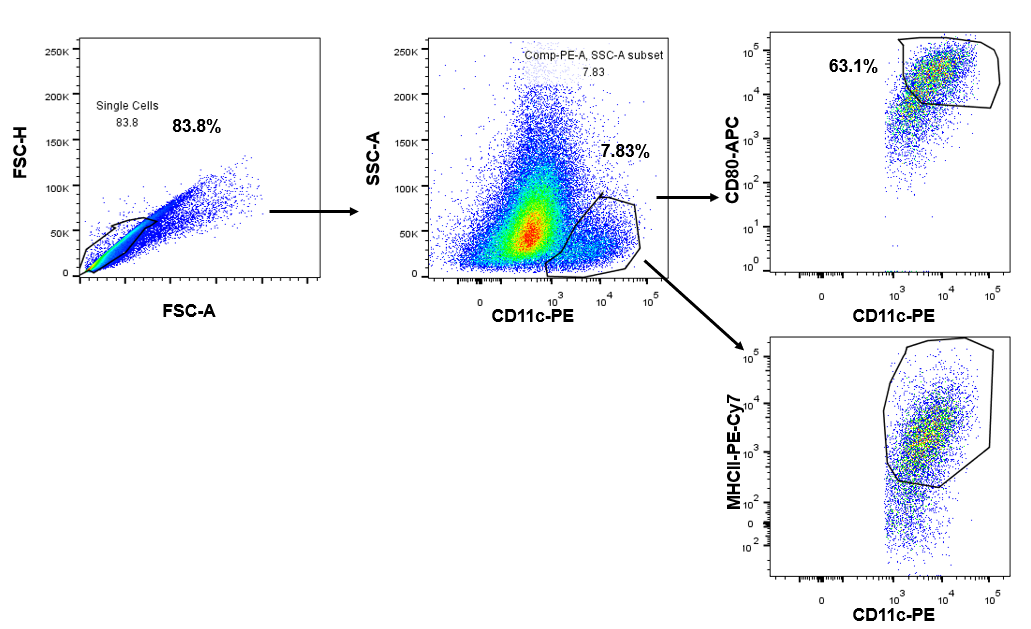


**Figure S6**. Data collection diagrams of flow cytometry for activated DCs (CD11c^+^CD80^+^ and CD11c^+^MHCII^+^) in MFC subcutaneous tumors after various treatments.

**
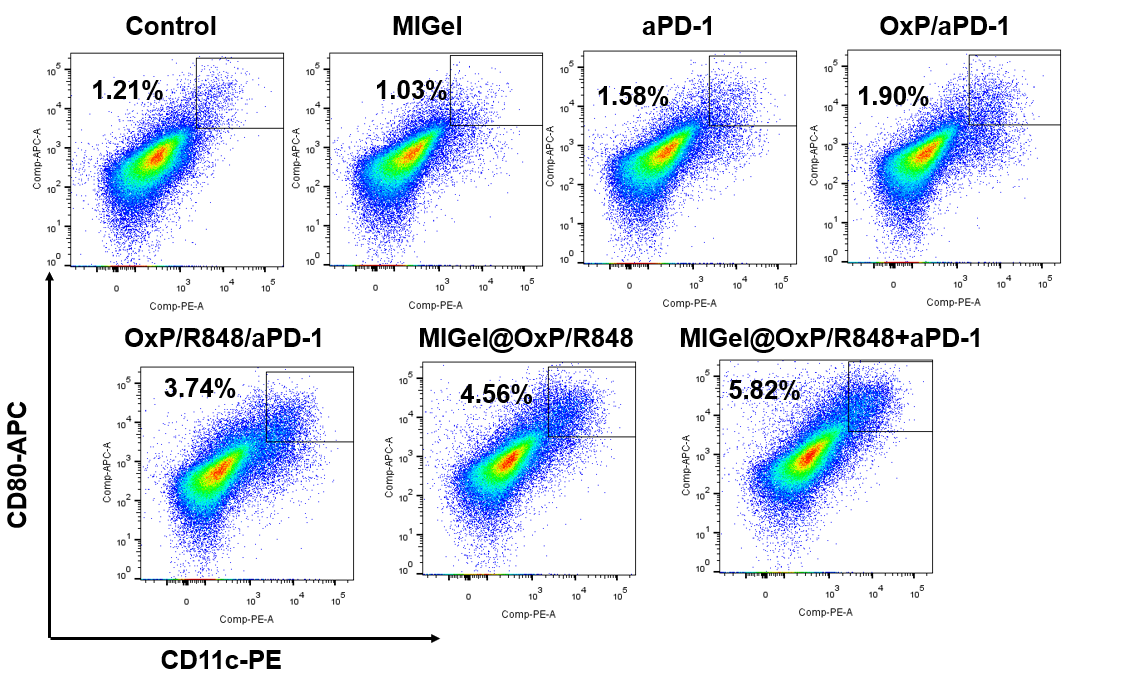
**

**Figure S7**. Representative scatter diagrams of activated DCs (CD11c^+^CD80^+^) cells in MFC subcutaneous tumors after various treatments.


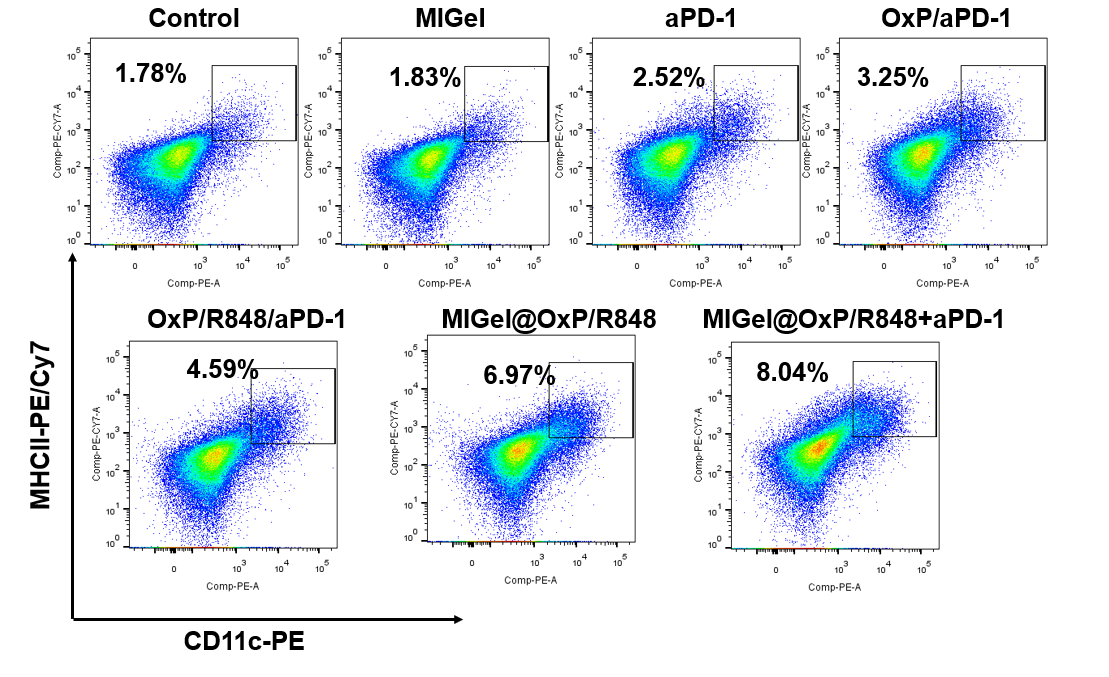


**Figure S8**. Representative scatter diagrams of activated DCs (CD11c^+^MHCII^+^) cells in MFC subcutaneous tumors after various treatments.


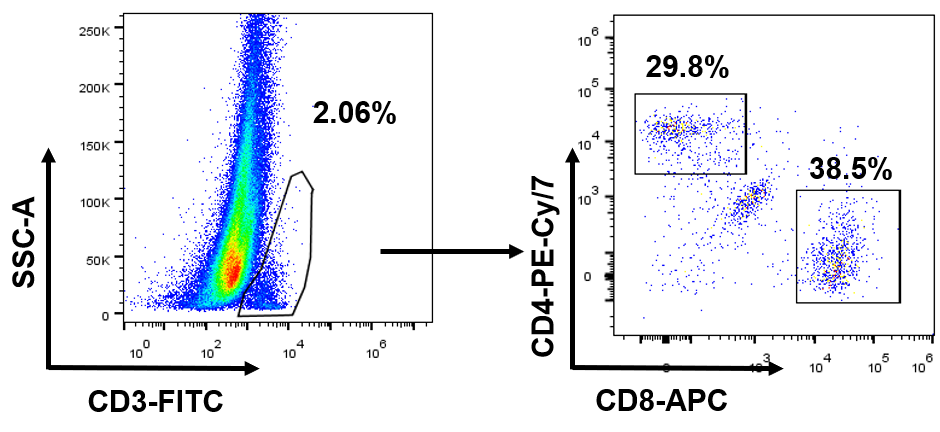


**Figure S9**. Data collection diagrams of flow cytometry for T cells in MFC subcutaneous tumors after various treatments.


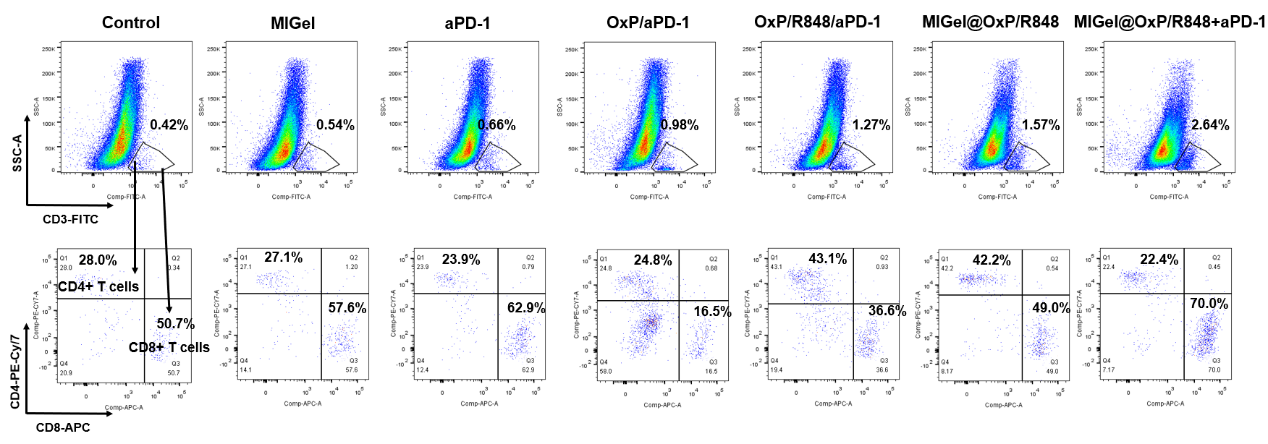


**Figure S10**. Representative scatter diagrams of CD3^+^, CD4^+^ and CD8^+^ T cells in MFC subcutaneous tumors after various treatments.


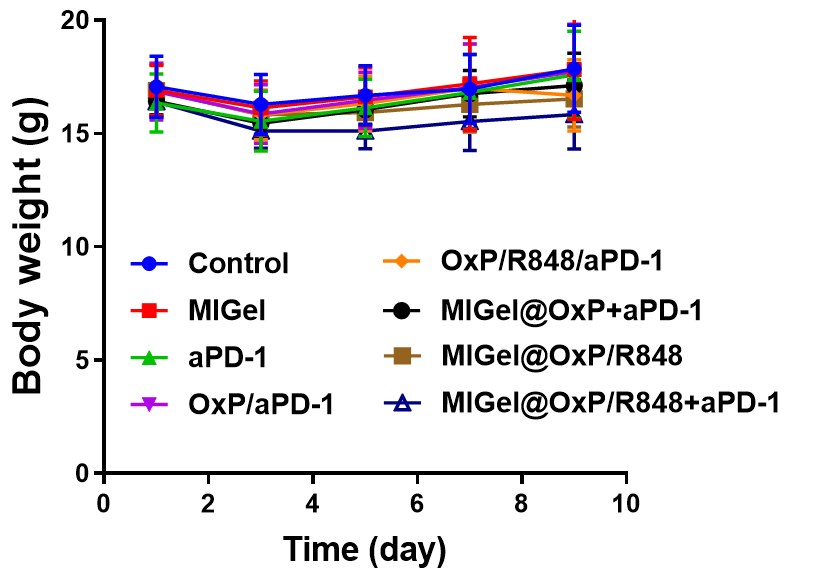


**Figure S11**. The change of body weights during the treatment period for MFC orthotopic tumor models after various treatments. *n* = 6.


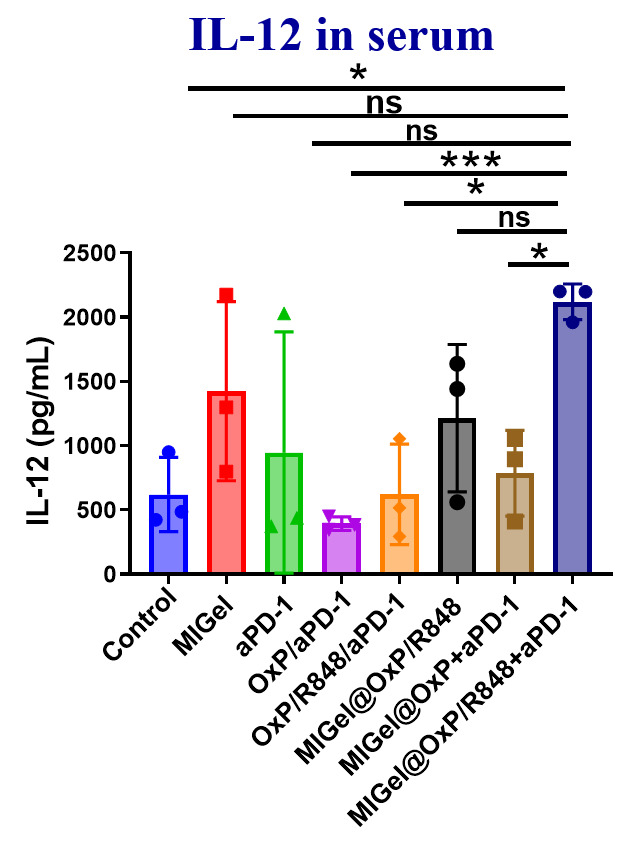


**Figure S12**. The level of IL-12 in the serum after various treatments for orthotopic gastric tumors at day 14. *n* = 3.


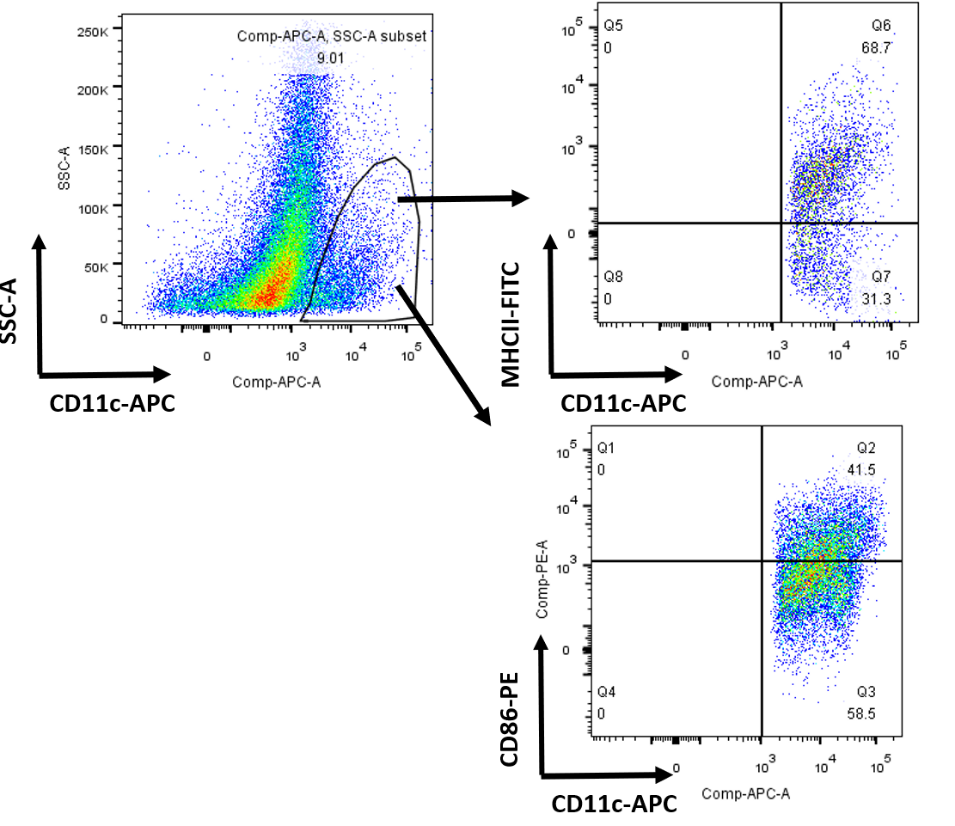


**Figure S13**. Data collection diagrams of flow cytometry for activated DCs in MFC orthotopic tumors after various treatments.


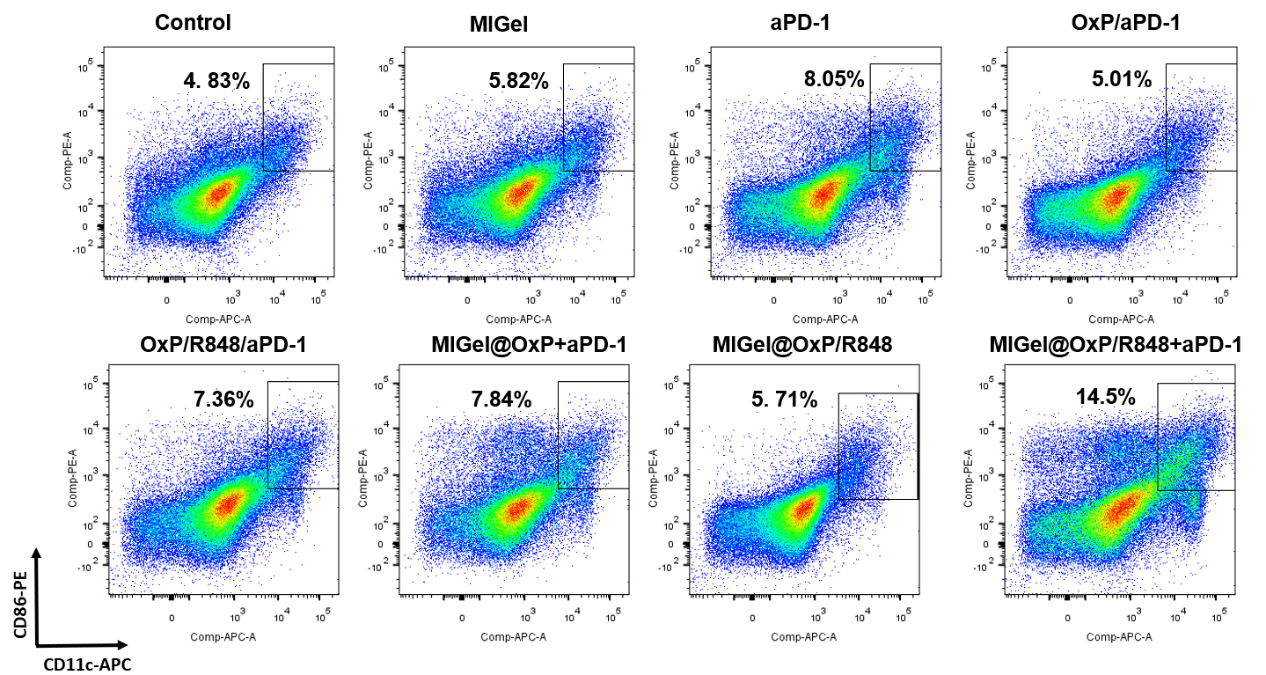


**Figure S14**. Representative scatter diagrams of activated DCs (CD11c^+^CD86^+^) in MFC orthotopic tumors after various treatments.


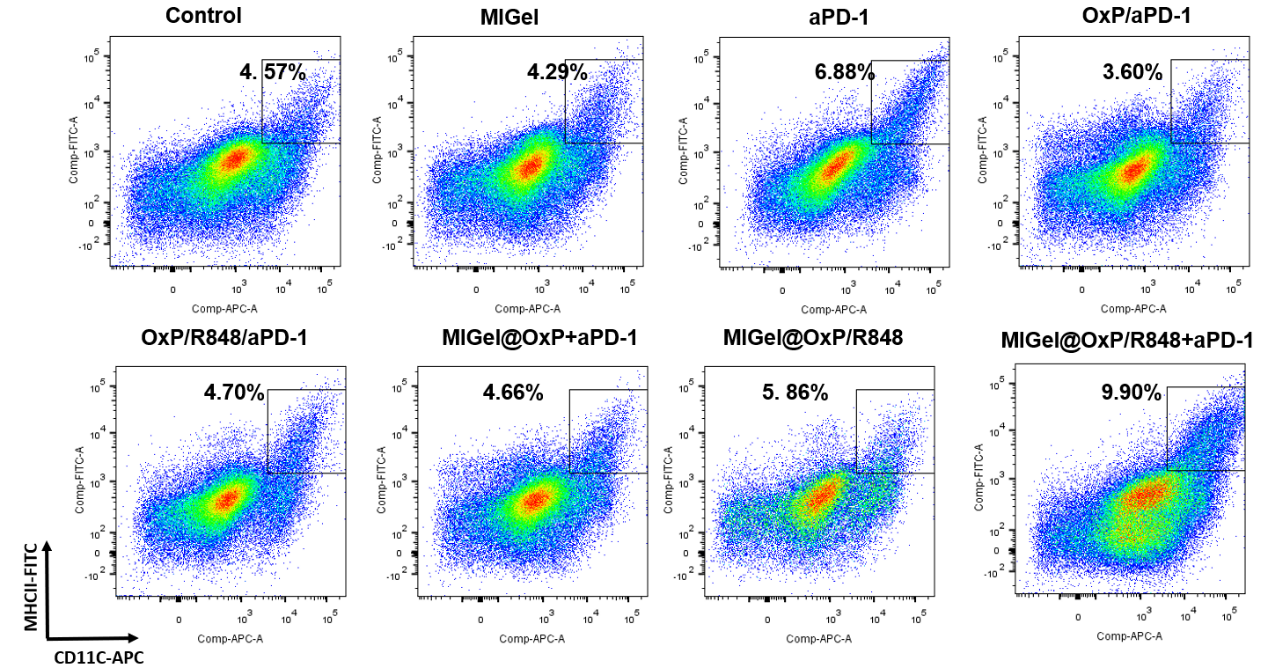


**Figure S15**. Representative scatter diagrams of activated DCs (CD11c^+^MHCII^+^) in MFC orthotopic tumors after various treatments.


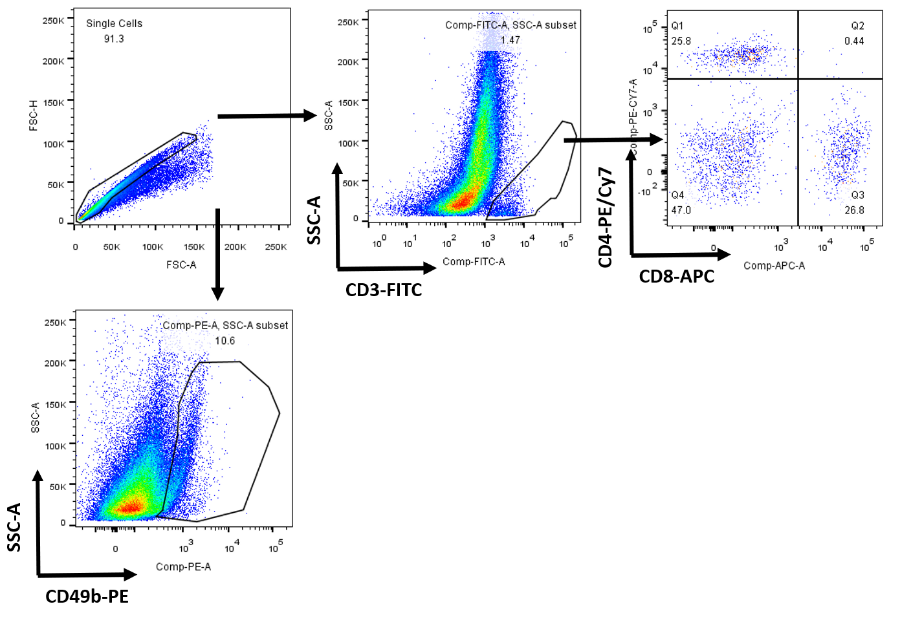


**Figure S16**. Data collection diagrams of flow cytometry for NK cells and T cells in MFC orthotopic tumors after various treatments.


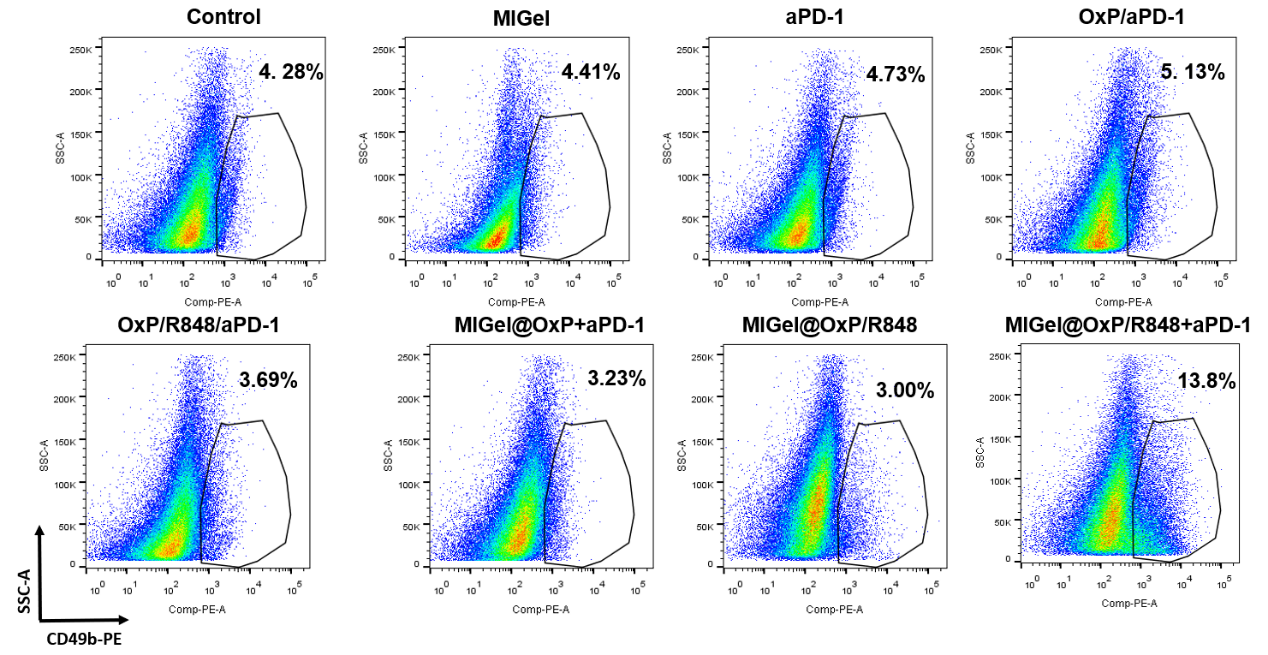


**Figure S17**. Representative scatter diagrams of NK cells in MFC orthotopic tumors after various treatments.


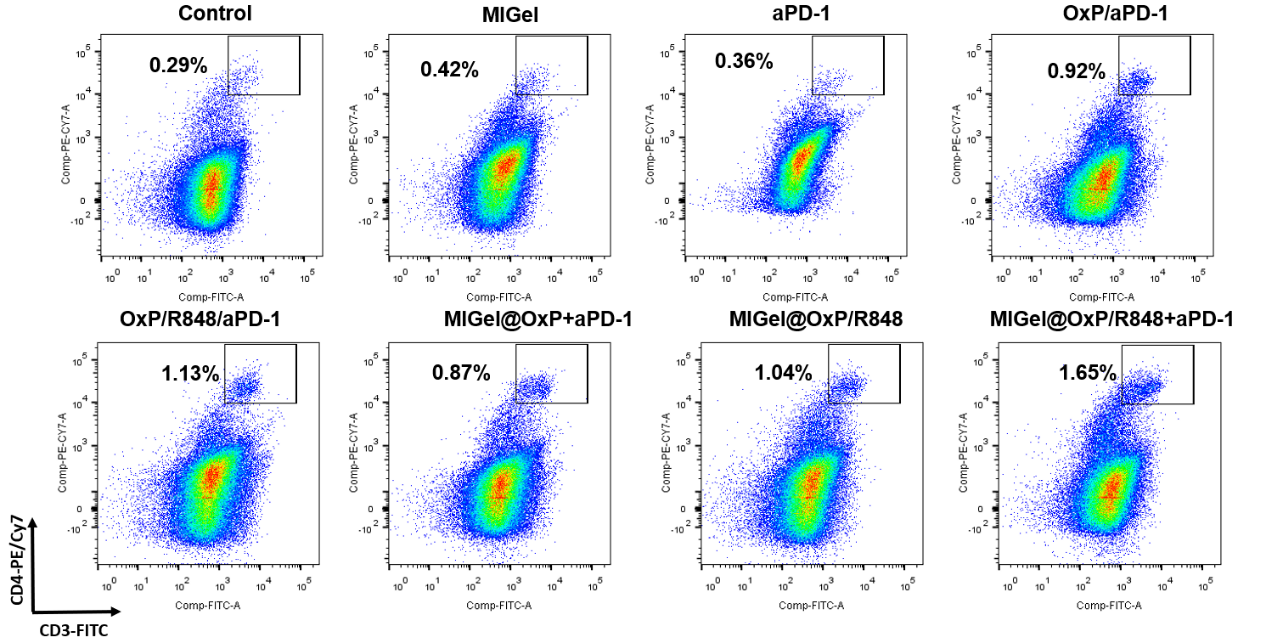


**Figure S18**. Representative scatter diagrams of CD4^+^ T cells in MFC orthotopic tumors after various treatments.


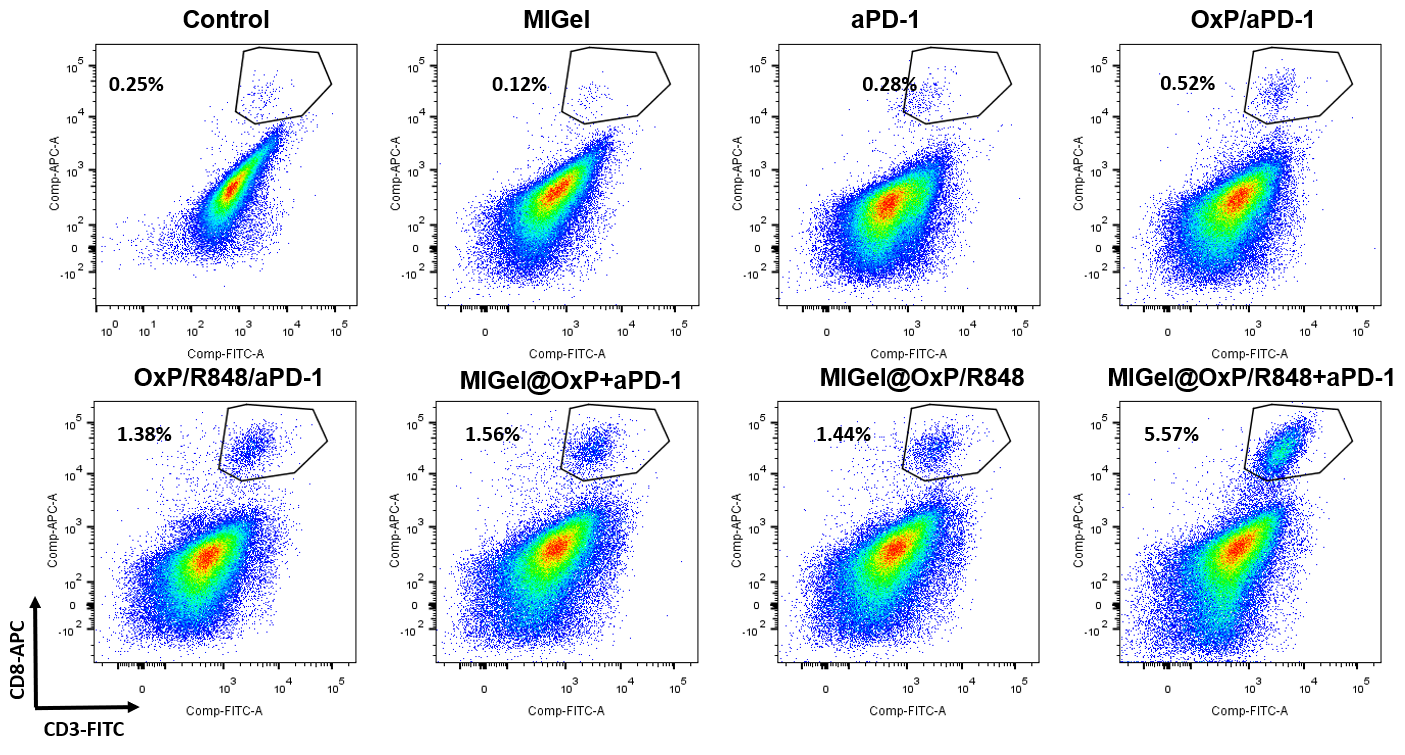


**Figure S19**. Representative scatter diagrams of CD8^+^ T cells in MFC orthotopic tumors after various treatments.


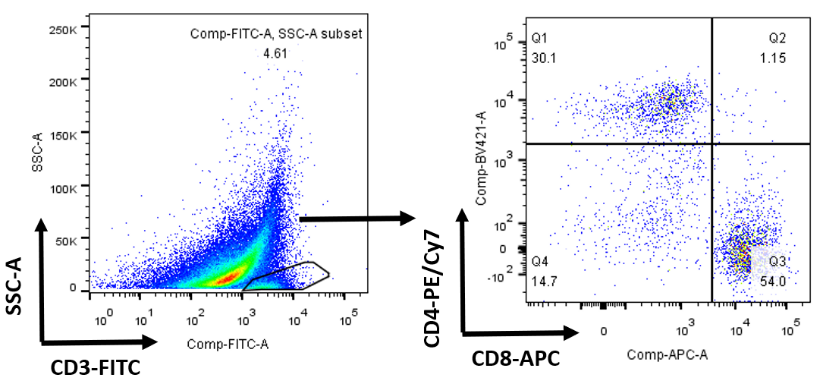


**Figure S20**. Data collection diagrams of flow cytometry for T cells in MFC liver metastasis tumors after various treatments.


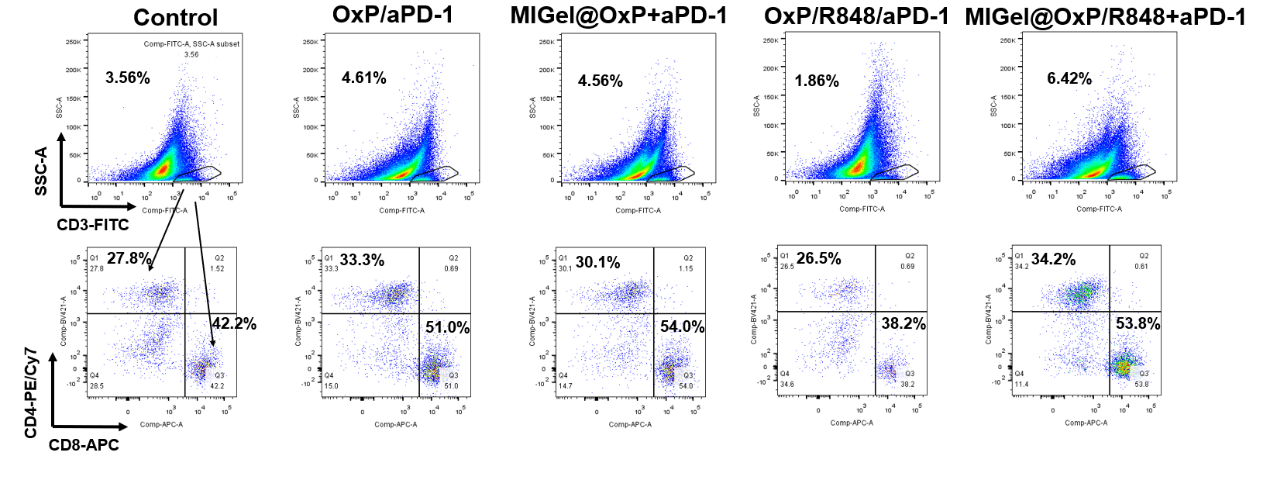


**Figure S21**. Representative scatter diagrams of CD4^+^ and CD8^+^ T cells in MFC liver metastasis tumors after various treatments.


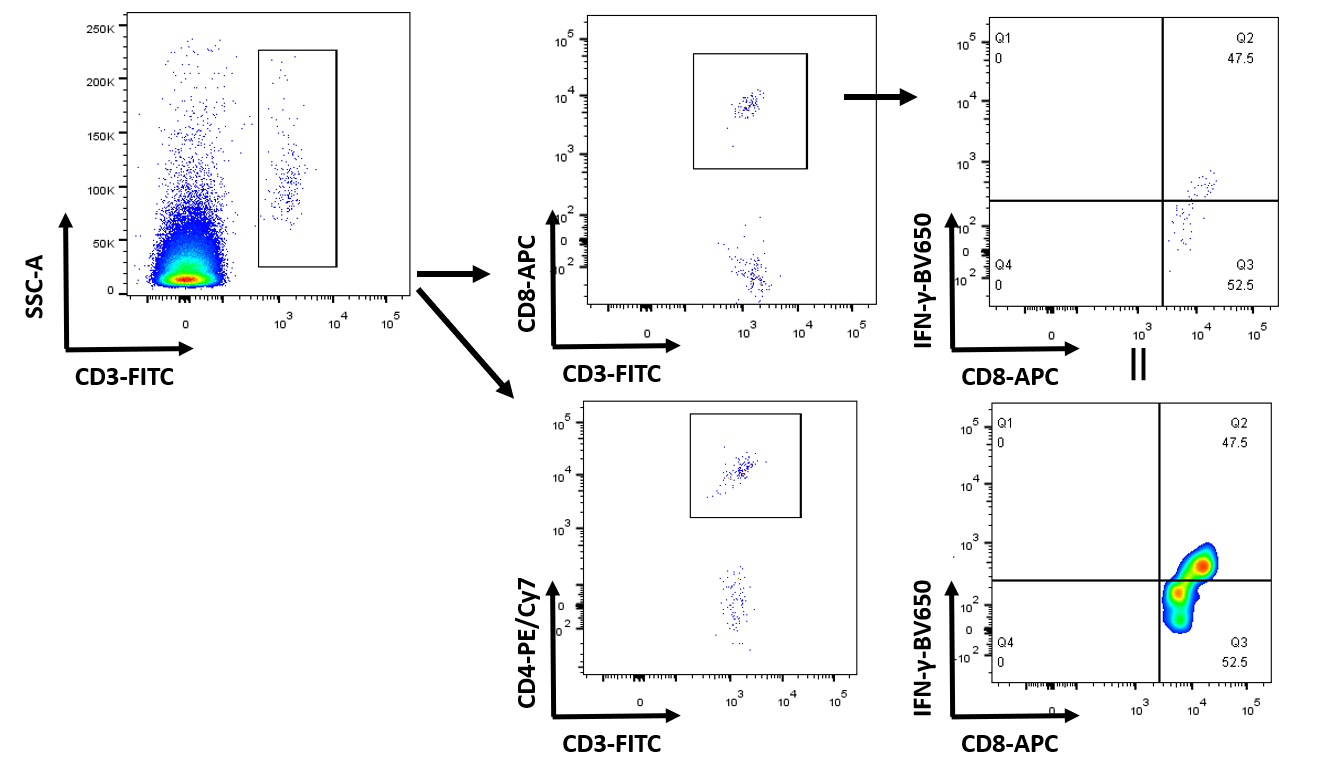


**Figure S22**. Data collection diagrams of flow cytometry for T cells in the blood after various treatments for MFC liver metastasis tumor models.


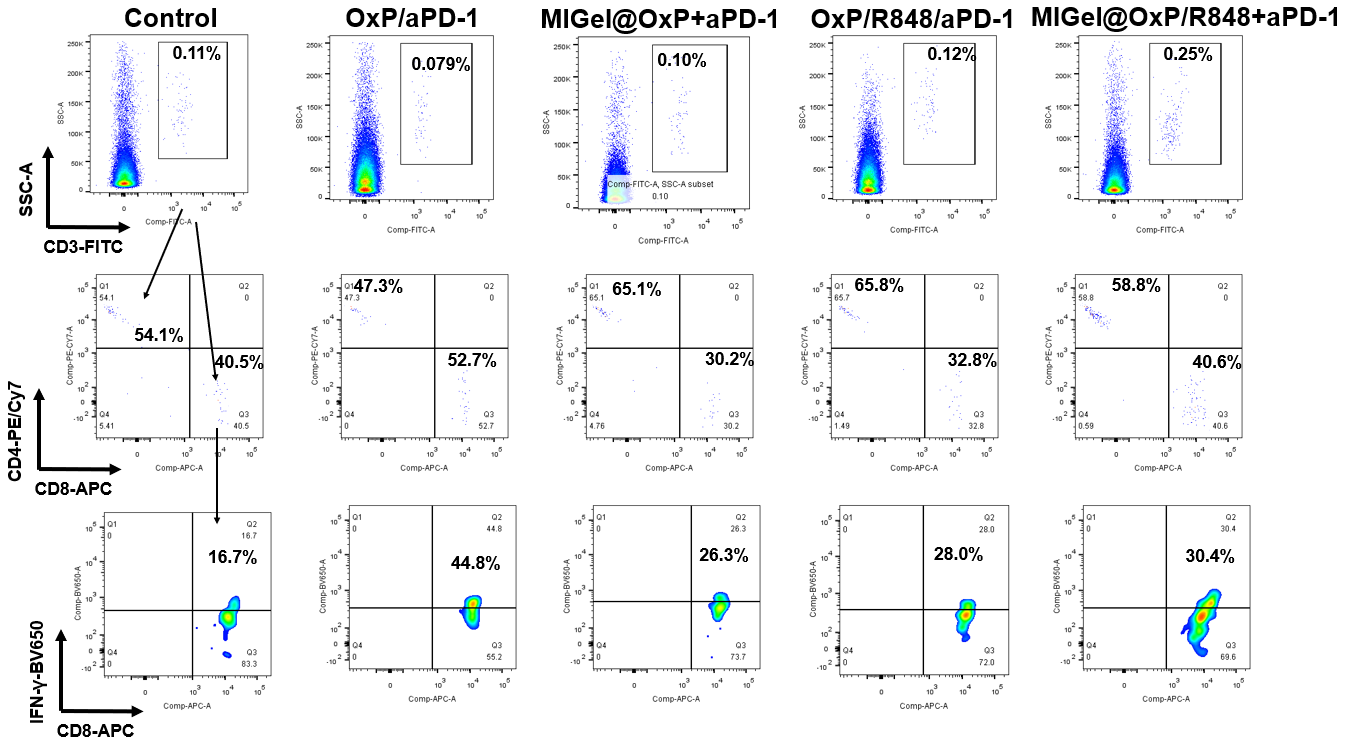


**Figure S23**. Representative scatter diagrams of CD4^+^, CD8^+^ and CD8^+^IFN-γ^+^ T cells in the blood after various treatments for MFC liver metastasis tumors models.


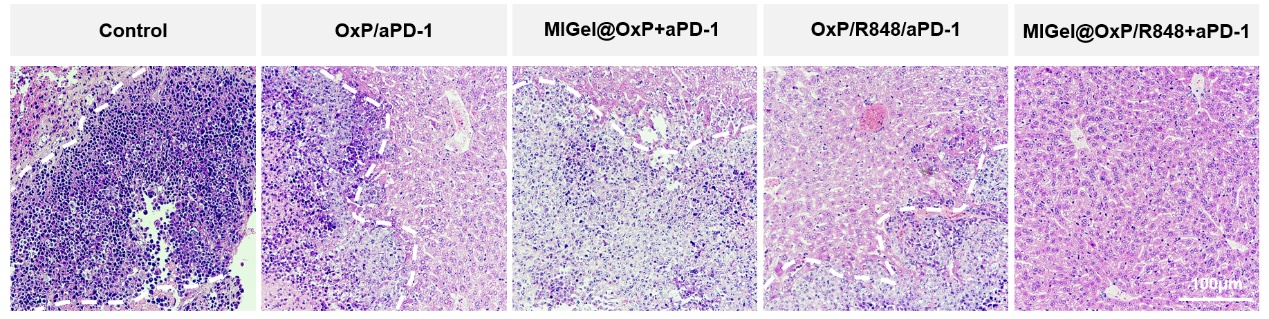


**Figure S24**. Representative HE staining images of orthotopic liver tumors after various treatments. The dashed area represents the tumors in the livers.


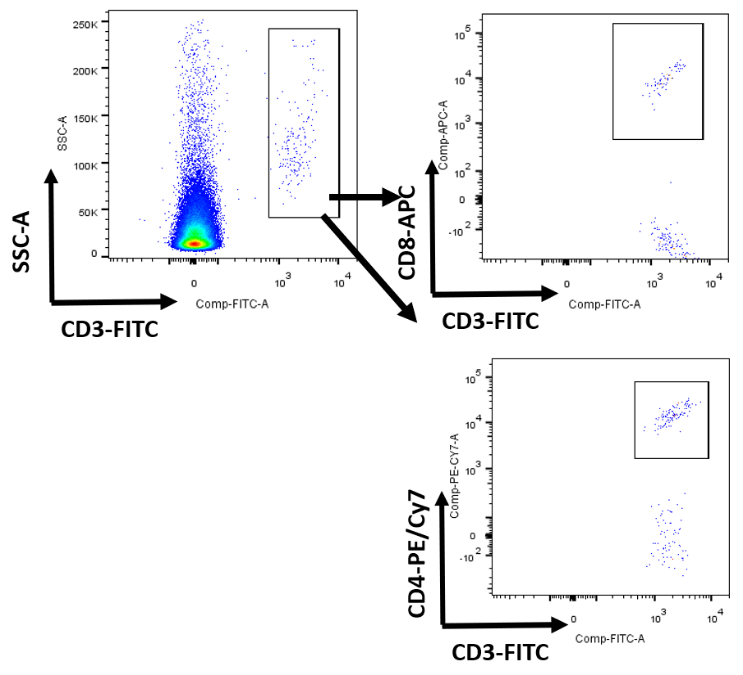


**Figure S25**. Data collection diagrams of flow cytometry for T cells in the blood after various treatments for H22 orthotopic live models.


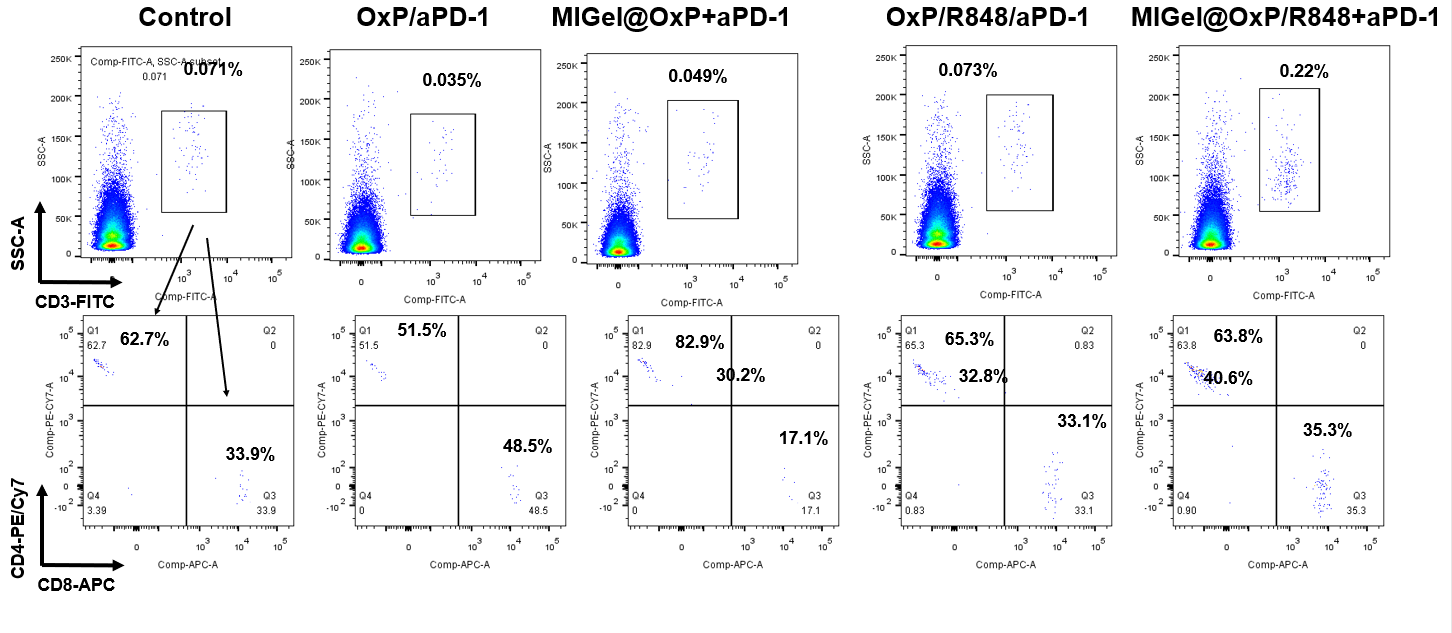


**Figure S26**. Representative scatter diagrams of CD4^+^ and CD8^+^ in the blood after various treatments for orthotopic H22 liver tumor models.


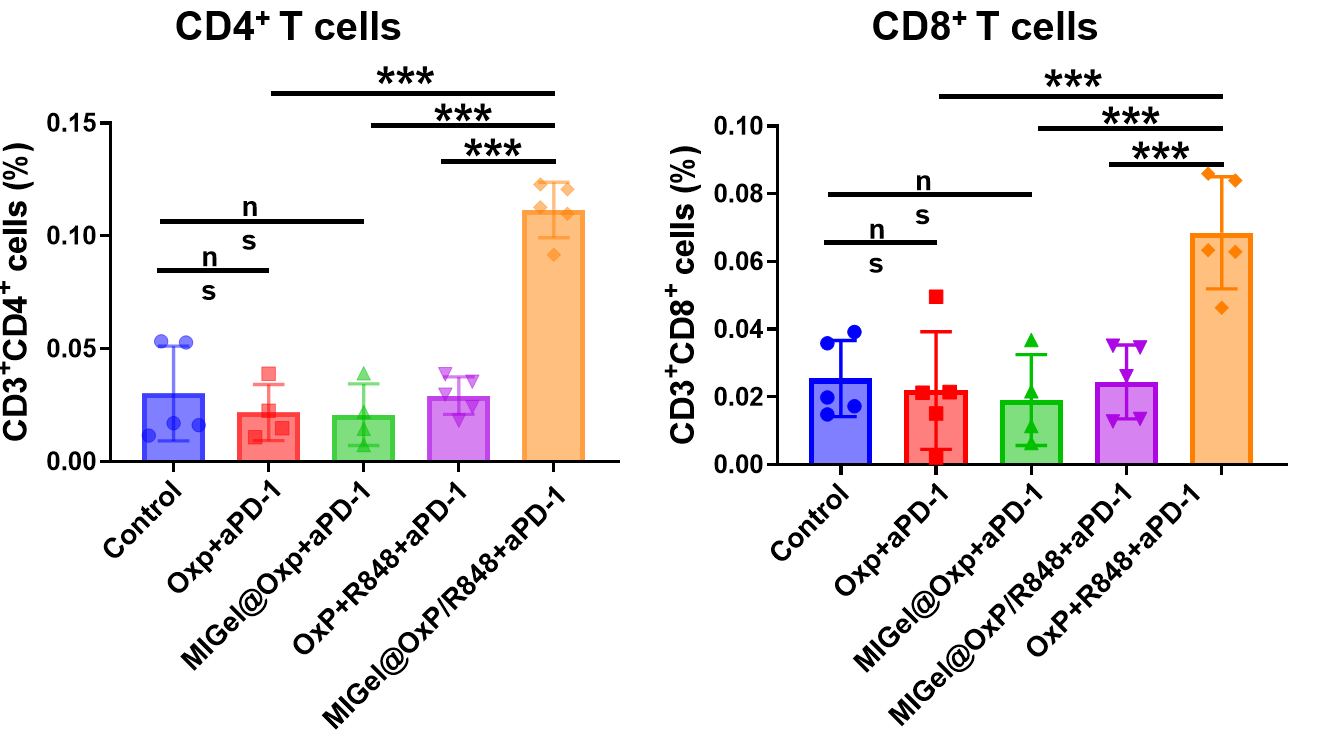


**Figure S27**. The proportions of CD4^+^ and CD8^+^ T cells in the blood of the mice after various treatments for orthotopic H22 liver tumor models. *n* = 5.

**Table S1.** Pharmacokinetic parameters estimated for free OxP/R848 and MIGel@OxP/R848 in rats.

| **Types** | **t_1/2_^a^ (h)** | **AUC_0–t_^b^**  **(μg/mL d)** | **CL^c^ (mL/d/kg)** | | **Vd^d^ (mL/kg)** |
| --- | --- | --- | --- | --- | --- |
| **Free OxP** | 0.64 ± 0.13 | 1.50 ± 0.51 | 285.99 ±21.35 | 891.77 ±21.35 | |
| **MIGel@OxP** | 3.81 ± 0.56 | 14.3 ± 1.91 | 10.86 ± 1.52 | 63.25 ± 2.28 | |
| **Free R848** | 0.49 ± 0.12 | 9.93 ± 1.34 | 100.23 ± 7.92 | 172.63 ± 9.41 | |
| **MIGel@R848** | 4.65 ± 0.67 | 34.9 ± 4.76 | 32.61 ± 4.31 | 50.65 ± 4.52 | |

^a^ t_1/2_: half-life.
^b^ AUC_0–t_: area under the drug concentration–time curve from 0 to 12 h in plasma.
^c^ CL: total body clearance.

^d^ V_d_: apparent volume of distribution

**Table S2.** **Antibodies used in the study.**

| Antibodies | Company | Catalog No. | Application | Dilution fold |
| --- | --- | --- | --- | --- |
| FITC Anti CD3 | BioLegend | 100204 | Flow | 1:400 |
| PE-Cy7 Anti-CD4 | BioLegend | 100422 | Flow | 1:400 |
| APC Anti-CD8 | BioLegend | 100712 | Flow | 1:400 |
| Alex Fluor 700-A Anti-CD8 | BioLegend | 100737 | Flow | 1:400 |
| PE Anti-CD11c | BioLegend | 117308 | Flow | 1:400 |
| APC-Cy7 Anti-MHCII | BioLegend | 116630 | Flow | 1:400 |
| APC-Cy7 Anti-CD80 | BioLegend | 104722 | Flow | 1:400 |
| APC Anti-CD80 | BioLegend | 104713 | Flow | 1:400 |
| PE-Cy7 Anti-CD86 | BioLegend | 105014 | Flow | 1:400 |
| APC Anti-CD11b | BioLegend | 101212 | Flow | 1:100 |
| FITC Anti-CD206 | BioLegend | 141704 | Flow | 1:400 |
| PE-Cy7 Anti-CD274 | BioLegend | 124314 | Flow | 1:400 |

**Table S3. Elisa kits used in the study.**


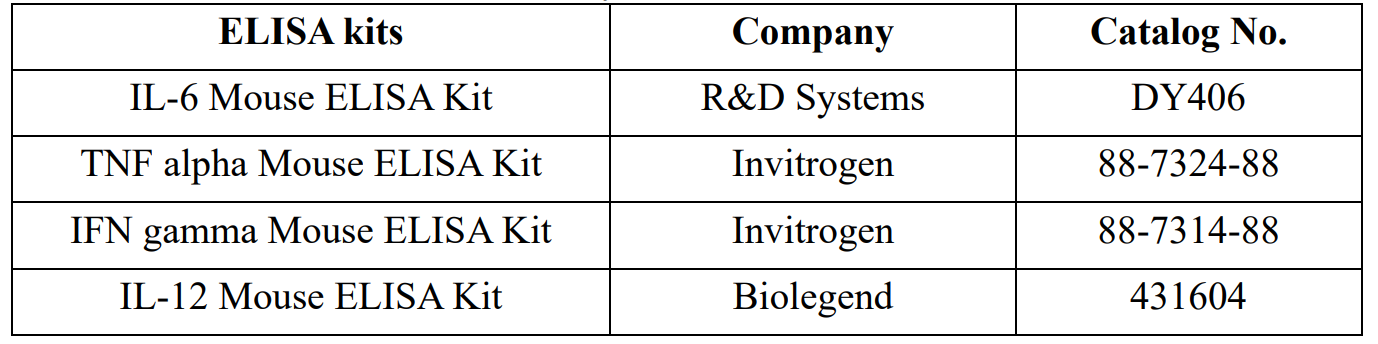


**Supplemental References**

[1] a)G. Ji, Y. Zhang, X. Si, H. Yao, S. Ma, Y. Xu, J. Zhao, C. Ma, C. He, Z. Tang, X. Fang, W. Song, X. Chen, Advanced Materials 2021, 33, 2004559; b)X. Si, G. Ji, S. Ma, Y. Xu, J. Zhao, Z. Huang, Y. Zhang, W. Song, Z. Tang, Acs Biomaterials Science & Engineering 2020, 6, 5281.

[2] Y. Zhang, M. Huo, J. Zhou, S. Xie, Computer Methods and Programs in Biomedicine 2010, 99, 306.
